# Incidence rate and predictors of COVID-19 in the two largest cities of Burkina Faso - prospective cohort study in 2021 (ANRS-COV13)

**DOI:** 10.1186/s12879-023-08361-2

**Published:** 2023-06-12

**Authors:** Nongodo Firmin Kaboré, Samiratou Ouédraogo, Ariane Kamga Mamguem, Isidore Tiandiogo Traoré, Dramane Kania, Hermann Badolo, Guillaume Sanou, Amariane Koné, Mimbouré Yara, Thérèse Kagoné, Esperance Ouédraogo, Blahima Konaté, Rachel Médah, Nathalie de Rekeneire, Armel Poda, Arnaud Eric Diendéré, Boukary Ouédraogo, Oumar Billa, Gilles Paradis, Tienhan Sandrine Dabakuyo-Yonli, Halidou Tinto

**Affiliations:** 1 Centre MURAZ, Institut National de Santé Publique (INSP), BP 390, Bobo-Dioulasso, Burkina Faso; 2 Observatoire national de la santé de la population (ONSP), Institut National de Santé Publique, Ouagadougou, Burkina Faso; 3 Institut National de Santé Publique du Québec (INSPQ), Montréal, Québec Canada; 4 The Department of Epidemiology, Biostatistics and Occupational Health, Faculty of Medicine, McGill University, Montreal, QC Canada; 5 Epidemiology and Quality of Life Research Unit, INSERM U1231, Georges Francois Leclerc Centre – UNICANCER, Dijon, France; 6 Institut Supérieur des Sciences de la Santé (INSSA), Université Nazi Boni (UNB), Bobo Dioulasso, Burkina Faso; 7 Centre National de Recherche et de Formation sur le Paludisme, Institut National de Santé Publique, Ouagadougou, Burkina Faso; 8 Département de médicine, pharmacopée traditionnelle et pharmacie, Institut de Recherche en Sciences de la Santé (IRSS) - Centre National de la Recherche Scientifique et Technologique (CNRST), Ouagadougou, Burkina Faso; 9 Département de Socio-Économie et d’Anthropologie du Développement (DSEAD), Institut des Sciences des Sociétés (INSS) - Centre National de la Recherche Scientifique et Technologique (CNRST), Ouagadougou, Burkina Faso; 10 Fondation Mérieux, Lyon, France; 11 Service des maladies infectieuses, Centre Hospitalier Universitaire Sourô Sanou, Bobo Dioulasso, Burkina Faso; 12 Centre Hospitalier Universitaire de Bogodogo, Ouagadougou, Burkina Faso; 13 Direction des systèmes d’information en santé (DSIS), ministère de la Santé et de l’Hygiène Publique, Ouagadougou, Burkina Faso; 14 Institut de Recherche en Sciences de la Santé (IRSS) - Unité de Recherche Clinique de Nanoro, Centre National de la Recherche Scientifique et Technologique (CNRST), Ouagadougou, Burkina Faso

**Keywords:** SARS-CoV-2, Sero-epidemiology, COVID-19 incidence, Africa, Burkina Faso

## Abstract

**Background:**

Early data on COVID-19 (based primarily on PCR testing) indicated a low burden in Sub-Saharan Africa. To better understand this, this study aimed to estimate the incidence rate and identify predictors of SARS-CoV-2 seroconversion in the two largest cities of Burkina Faso. This study is part of the EmulCOVID-19 project (ANRS-COV13).

**Methods:**

Our study utilized the WHO Unity protocol for cohort sero-epidemiological studies of COVID-19 in general population. We conducted random sampling stratified by age group and sex. Individuals aged 10 years and older in the cities of Ouagadougou and Bobo-Dioulasso, Burkina Faso were included and surveyed at 4 time points, each 21 days apart, from March 3 to May 15, 2021. WANTAI SARS-CoV-2 Ab ELISA serological tests were used to detect total antibodies (IgM, IgG) in serum. Predictors were investigated using Cox proportional hazards regression.

**Results:**

We analyzed the data from 1399 participants (1051 in Ouagadougou, 348 in Bobo-Dioulasso) who were SARS-CoV-2 seronegative at baseline and had at least one follow-up visit. The incidence rate of SARS-CoV-2 seroconversion was 14.3 cases [95%CI 13.3–15.4] per 100 person-weeks. The incidence rate was almost three times higher in Ouagadougou than in Bobo-Dioulasso (Incidence rate ratio: IRR = 2.7 [2.2–3.2], p < 0.001). The highest incidence rate was reported among women aged 19–59 years in Ouagadougou (22.8 cases [19.6–26.4] per 100 person-weeks) and the lowest among participants aged 60 years and over in Bobo-Dioulasso, 6.3 cases [4.6–8.6] per 100 person-weeks. Multivariable analysis showed that participants aged 19 years and older were almost twice as likely to seroconvert during the study period compared with those aged 10 to 18 years (Hazard ratio: HR = 1.7 [1.3–2.3], p < 0.001). Those aged 10–18 years exhibited more asymptomatic forms than those aged 19 years and older, among those who achieved seroconversion (72.9% vs. 40.4%, p < 0.001).

**Conclusion:**

The spread of COVID-19 is more rapid in adults and in large cities. Strategies to control this pandemic in Burkina Faso, must take this into account. Adults living in large cities should be the priority targets for vaccination efforts against COVID-19.

**Supplementary Information:**

The online version contains supplementary material available at 10.1186/s12879-023-08361-2.

## Introduction

At the beginning of the COVID-19 pandemic, and before the widespread use of serological tests, statistics indicated a low burden of infection in Africa compared to other continents [1, 2]. SARS-COV-2 (the virus responsible for COVID-19) infection was diagnosed or screened by detection of viral particles by PCR testing on nasopharyngeal swabs. However, given the limited resources in African countries, the weakness of their health systems and the high cost of PCR testing, many experts have attributed the low number of COVID-19 cases in Africa to under-diagnosis/screening [3, 4]. This raises the question of the true extent of the spread of SARS-COV-2 infection in Africa. To better understand the true burden and distribution of disease, the World Health Organization (WHO) has encouraged all countries to conduct seroprevalence studies on COVID-19. Unlike viral particles, which persist beyond one month in the upper respiratory tract in less than half of infected persons, serological markers (antibodies) of SARS-CoV-2 infection remain in the blood for a longer period of time, and thus provide a better means of estimating the extent of infection in a population by allowing measurement of cumulative incidence [5–8]. Early studies based on serological testing indicated a similar level of infection across continents [9]. However, most of these sero-epidemiological studies were cross-sectional surveys and precluded an assessment of the dynamics (incidence) of the epidemic over time. In order to better assess the dynamics of the COVID-19 pandemic in the different segments of the population, the WHO proposed the use of a standardized Unity protocol for sero-epidemiological studies in the general population [10]. We used this Unity protocol to investigate the incidence rate and predictors of SARS-CoV-2 seroconversion in the two largest cities of Burkina Faso, one year after the official declaration of the first case of COVID-19 in the country. This study was conducted within the framework of the ANRS-COV13 project (EmulCOVID-19) [11].

## Methods

### Study design

This was a prospective observational cohort study, conducted as part of a multidisciplinary research study on COVID-19 in Burkina Faso (EmulCOVID-19 - ANRS-COV13) [11]. Data were collected over the period from March 3 to May 15, 2021. The study was conducted as a household survey in the two largest cities of Burkina Faso, namely Ouagadougou, the largest city in the country, and the political capital, and Bobo-Dioulasso, which is the second largest city in the country and the economic capital. The population of Burkina Faso was estimated to be 21.5 million inhabitants in 2021, with Ouagadougou’s population estimated at about 2.7 million and Bobo-Dioulasso’s at about 900,000 [12].

### Study population and sampling

The study population comprised individuals aged 10 years and older, residing in selected households in Ouagadougou and Bobo-Dioulasso.

For EmulCOVID-19, following the methodology provided by WHO for COVID-19 sero-epidemiological studies in the general population, we conducted random sampling, stratified by age group and sex. Full detail of the study sampling method have previously been described in the study protocol [11]. Briefly, we used a two-stage sampling strategy: first, we randomly selected 168 Enumeration Areas (EA) in both cities with a probability proportional to the total number of EA in each city: 111 EA in Ouagadougou and 57 EA in Bodo Dioulasso. Our survey team then conducted a census of all living individuals in the households located in these EA. The second sampling stage consisted in random selection of long-term residents aged 10 years and older from each sampled EA, stratifying by age group (10–14 years-old, 14–18 years-old, 19–50 years-old, 60 years and older) and sex (male, female), and inviting them to provide a blood sample and complete a questionnaire.

For this analysis, we included participants included in EmulCOVID-19 who were seronegative at baseline survey and had at least one follow-up visit.

### Serological test for SARS-CoV-2

For this study, the WANTAI SARS-CoV-2 Ab ELISA serological tests were used [13]. These are ELISAs that detect total antibodies (IgM, IgG) to SARS-CoV-2 in serum. According to the manufacturer, the sensitivity and specificity of these tests are 94.36% and 100% respectively [13]. The tests were donated to the research team by WHO.

### Study variables

- Seroconversion was asserted whenever anti-SARS-CoV2 antibodies were detected in a participant who did not have antibodies at baseline. In this study, we approximated the cumulative incidence of COVID-19 by the rate of seroconversion to SARS-CoV-2, i.e. the number of seronegative participants at baseline who subsequently became positive during follow-up. The incidence rate of SARS-CoV-2 seroconversion was calculated as the number of cases per 100 person-weeks.
- The sociodemographic characteristics considered in our analysis were: Age (10–14 / 15–18 / 19–29 / 30–59 / 60 years old), sex (female / male), educational level (not enrolled in school / primary-literate / secondary / university), city of residence (Ouagadougou / Bobo-Dioulasso), marital status (single / couple), and main occupation during the past 12 months (trader / artisan / housewife / unemployed / student / other).
- Exposure to SARS-CoV-2 was reported if in the 14 days prior to data collection, the participant had been exposed to COVID-19 positive individuals, worked with COVID-19 positive individuals, been within one meter of a COVID-19 positive individual, shared the same enclosed environment as a COVID-19 positive individual (including sharing a classroom or household or being at the same gathering), traveled (bus / taxi / personal car / plane) with a COVID-19 positive individual, or provided direct care to COVID-19 positive individuals (see questionnaire in Additional file 1).
- The presence of COVID-19 symptoms in the previous 14 days was established by the presence of at least one of the symptoms listed in the questionnaire (Additional file 1). These symptoms included fever (temperature > 38 °C), cold, cough / sneezing, headache, breathlessness, loss of taste, loss of smell.

### Data collection

Adult participants (males 19–59 years-old, females 19–59 years-old, participants of 60 years-old and over) were surveyed at 4 time points each 21 days apart. At each visit, a blood sample was collected. Sociodemographic and clinical data as well as exposure factors were also collected at each of the four measurement times, using a questionnaire. Participants under 19 years-old (10–14 years-old, 15–18 years-old) were surveyed at the last two visits only. During the first two rounds, the research team carefully sensitized the parents (≥19 years-old) and obtain their consent before proceeding with the inclusion of children (< 19 years-old). The questionnaires did not include the names of the participants, but only the assigned study identification numbers. These questionnaires were recorded in a confidential database that could only be accessed by the research team.

### Statistical analysis

Categorical variables are presented as number and proportions and were compared using the Chi-2 or Fisher’s exact tests. The incidence rate of seroconversion was estimated by considering the effective follow-up time of each participant to his/her date of seroconversion or, in case of no seroconversion, to the date of the last visit (right censoring). The exact date of seroconversion was unknown, as it occurred between two survey timepoints. For this purpose, we considered the midpoint of the interval between two consecutive surveys as the date of seroconversion to calculate the incidence rates. Incidence rates by socio-demographic characteristics were compared using Mantel-Haenszel rate ratios (using the “stmh” command in Stata). Cumulative incidence curves by city were compared using Wilcoxon tests. We used multiple imputation using chained equations to handle missing data and we performed sensitivity analyses [14, 15]. The details of the imputation process and sensitivity analyses are given in Additional file 3. We investigated predictive factors for seroconversion using Cox proportional hazards regression [16]. In the interim analyses, we found that there was no difference in risk between 10-14-year-olds and 15-18-year-olds; we therefore pooled the two youth modalities into one. Bivariable analysis consisted of testing the contribution of each of the sociodemographic characteristic variables (age: 10–18/19–29/30–59/60 years-old, sex: female/male, educational level: not enrolled in school/primary-literate/secondary/university, marital status: single/coupled/not applicable, main occupation: trader-artisan/housewife-unemployed/student/other/missing data) to the occurrence of SARS-CoV-2 seroconversion. All variables that were associated with seroconversion with a p-value < 20%, by bivariable analysis, were included in the multivariable analysis. A top-down stepwise procedure was used to determine the final model.

In the subgroup of seroconverting participants, the proportion of asymptomatic patients aged 19 years and over was compared with that observed in participants aged 10–18 years. Statistical analyses were performed using Stata 15.1 software (StataCorp, Texas, USA) and the significance level for all statistical tests was 5%.

## Results

**Fig. 1:**
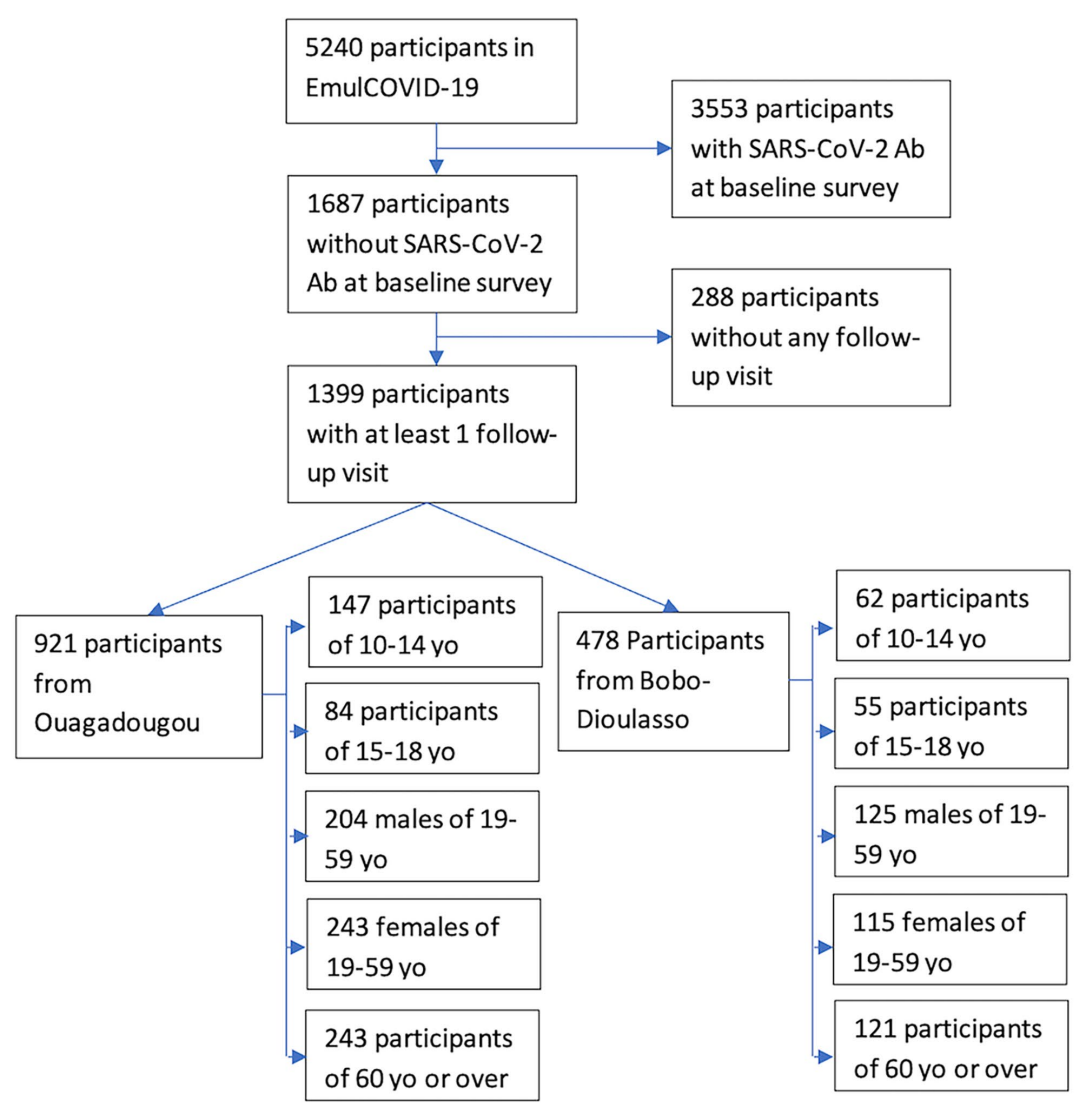
Flowchart of participants included in the analyses Yo: years-old; Ab: Antibodies

**Fig. 2:**
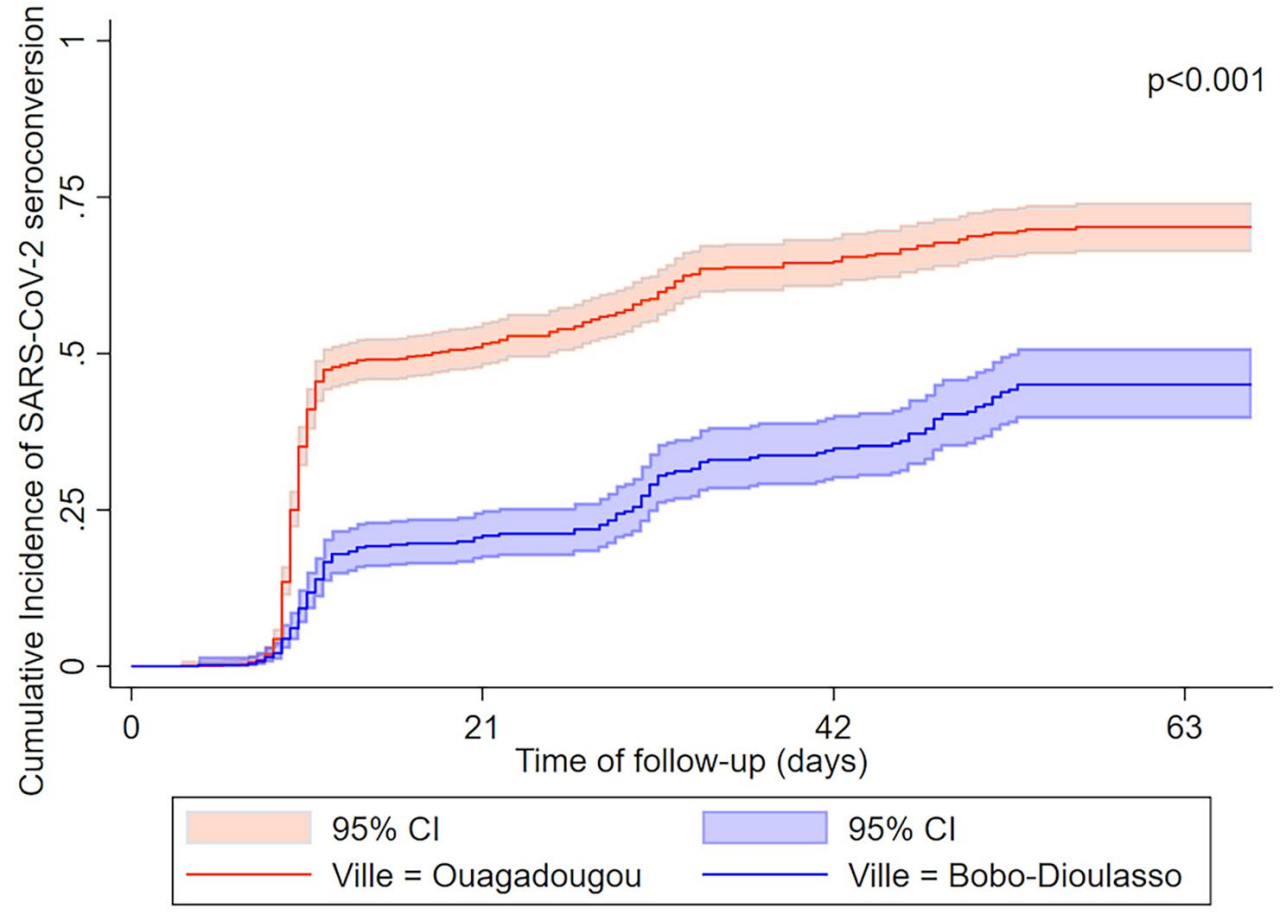
Kaplan-Meier curves for cumulative incidence of SARS-CoV-2 seroconversion by city of residence CI: Confidence interval

**Fig. 3:**
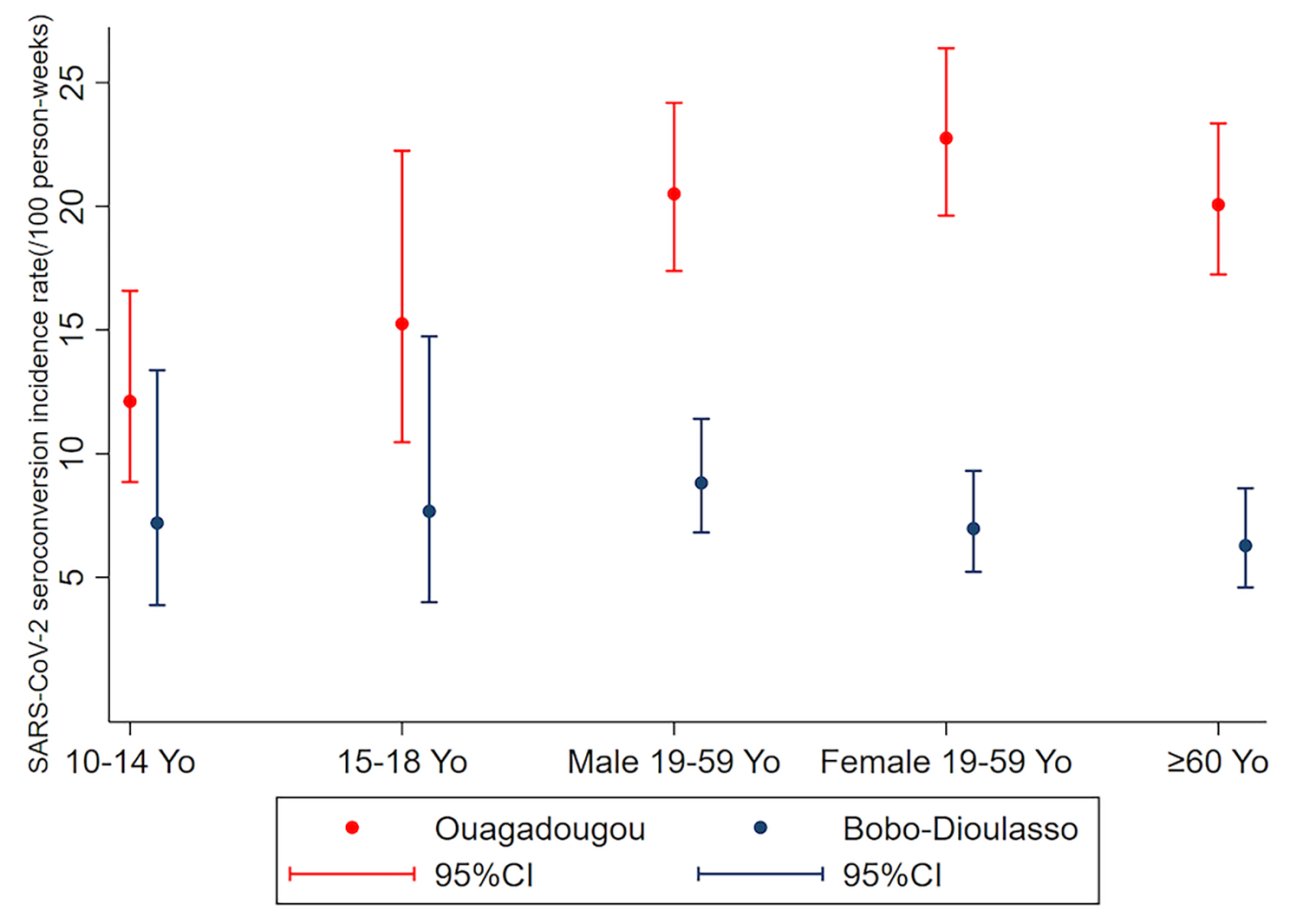
Incidence rates (per 100 person-weeks) of SARS-CoV-2 seroconversion by city and population group Yo: years-old; CI: Confidence Interval

Among 5240 individuals who participated in EmulCOVID-19, 3553 (67.8%) had positive SARS-CoV-2 serology at the baseline survey (D0). Of the 1687 participants who had negative serology at D0, 288 did not have any follow-up visit (Table S1 in Additional file 2). Our analyses included the data from the 1399 participants who were seronegative at D0 and had at least one follow-up visit (Fig. 1). The sociodemographic characteristics of these participants are presented in Table 1. The number of participants per survey (D0, D21, D42, D63) and per variable are presented in Additional file 2 (Table S2). The total number of cases of SARS-CoV-2 seroconversion during the follow-up was 711 (50.8% of participants). The proportion of participants aged 19 years or over who left the study before the D63 survey was 31.8% (334/1051). Of these, 50% had not seroconverted (Table S3 in Additional file 2). No participants reported receiving a COVID-19 vaccine during follow-up. The overall incidence rate of SARS-CoV-2 seroconversion was 14.3 cases [13.3–15.4] per 100 person-weeks. It was 19.7 cases [18.1–21.4] per 100 person-weeks in Ouagadougou, and 7.4 cases [6.3–8.6] per 100 person-weeks in Bobo-Dioulasso (Incidence rate ratio: IRR = 2.7 [95% confidence interval (CI) 2.2–3.2], p < 0.001) (Table 2; Fig. 2). The incidence rate of SARS-CoV-2 seroconversion was lower among children aged 10–18 years than in those aged 19 years and older (11.3 [CI 9.1–13.9] vs. 14.8 [13.7–16.0] cases per 100 person-weeks, p = 0.017) (Table 2). The highest incidence rate was reported among women aged 19–59 years in Ouagadougou (22.8 cases [19.6–26.4] per 100 person-weeks) and the lowest among participants aged 60 years and over in Bobo-Dioulasso (6.3 cases [4.6–8.6] per 100 person-weeks) (Fig. 3).

**Table 1:** Sociodemographic characteristics of participants at enrollment

Age group	≥ 19 yo, n(%)	10–18 yo, n(%)	Total, n(%)
**Number of participants**	1051	348	1399
**City of residence**			
Ouagadougou	690 (65.7)	231 (66.4)	921 (65.8)
Bobo-Dioulasso	361 (34.3)	117 (33.6)	478 (34.2)
**Population group**			
10–14 yo	---	209 (60.1)	209 (14.9)
15–18 yo	---	139 (39.9)	139 (9.9)
Male 19–59 yo	329 (31.3)	---	329 (23.5)
Female 19–59 yo	358 (34.1)	---	358 (25.6)
≥ 60 yo	364 (34.6)	---	364 (26.0)
**Sex**			
Male	511 (48.6)	132 (37.9)	643 (46.0)
Female	540 (51.4)	216 (62.1)	756 (54.0)
**Age (years)**			
10–18	---	348 (100)	348 (24.9)
19–29	274 (26.1)	---	274 (19.6)
30–59	413 (39.3)	---	413 (29.5)
≥ 60	364 (34.6)	---	364 (26.0)
**Educational level**			
Not enrolled in school	332 (31.6)	30 (8.6)	362 (25.9)
Literate/primary	287 (27.3)	150 (43.1)	437 (31.2)
Secondary	326 (31.0)	148 (42.5)	474 (33.9)
University	106 (10.1)	0	106 (7.6)
Missing data	0	20 (5.8)	20 (1.4)
**Marital status**			
Single	383 (36.4)	---	383 (27.4)
Couple	668 (65.6)	---	668 (47.7)
Not applicable	---	348 (100)	348 (24.9)
**Main occupation during the past 12 months**			
Trader/artisan	380 (36.2)	7 (2.0)	387 (27.7)
Housewife/unemployed	290 (27.6)	4 (1.2)	294 (21.0)
Student	129 (51.7)	180 (51.7)	309 (22.1)
Other	250 (23.8)	1 (0.3)	251 (17.9)
Missing data	2 (0.2)	156 (44.8)	158 (11.3)

Yo : years-old

**Table 2:** Incidence rate of SARS-CoV-2 seroconversion by sociodemographic characteristics of participants

Age group		≥ 19 yo			10–18 yo			Total		
		Incidence rate	95%CI	p-value	Incidence rate	95%CI	p-value	Incidence rate	95%CI	p-value
**Ouagadougou + Bobo-Dioulasso**		14.8	[13.7–16.0]	---	11.3	[9.1–13.9]	---	14.3	[13.3–15.4]	---
**City of residence**	Ouagadougou	21.1	[19.3–23.1]	< 0.001	13.2	[10.4–16.8]	0.024	19.7	[18.1–21.4]	< 0.001
	Bobo-Dioulasso	7.4	[6.3–8.7]		7.4	[4.7–11.6]		7.4	[6.3–8.6]	
**Population group**	10–14 yo	---	---	---	10.6	[8.0-14.1]	0.523	10.6	[8.0-14.1]	0.178
	15–18 yo	---	---	---	12.2	[8.8–17.0]		12.2	[8.8–17.0]	
	Male 19–59 yo	14.8	[12.9–17.0]	0.369	---	---	---	14.8	[12.9–17.0]	
	Female 19–59 yo	15.5	[13.6–17.6]		---	---	---	15.5	[13.6–17.6]	
	≥ 60 yo	14.2	[12.4–16.3]		---	---	---	14.2	[12.4–16.3]	
**Sex**	Male	14.7	[13.1–16.5]	0.852	11.5	[8.2 -16.2]	0.856	14.3	[12.9–15.9]	0.947
	Female	14.9	[13.4–16.6]		11.1	[8.4–14.5]		14.2	[12.9–15.7]	
**Age (years)**	10–18	---	---	---	11.3	[9.1–13.9]	---	11.3	[9.1–13.9]	0.132
	19–29	14.5	[12.4–16.9]	0.761	---	---	---	14.5	[12.4–16.9]	
	30–59	15.6	[13.8–17.6]		---	---	---	15.6	[13.8–17.6]	
	≥ 60	14.2	[12.4–16.3]		---	---	---	14.2	[12.4–16.3]	
**Educational level**	Not enrolled in school	14.7	[12.8–17.0]	0.967	10.5	[5.0–22.0]	0.997	14.5	[12.7–16.7]	0.943
	Literate/primary	14.2	[12.2–16.5]		11.3	[8.2–15.6]		13.5	[11.8–15.5]	
	Secondary	16.0	[14.0-18.3]		11.0	[9.9–15.3]		15.0	[13.3–17.0]	
	University	13.2	[10.3–16.9]		---	---	---	13.2	[10.3–16.9]	
**Marital status**	Single	14.7	[12.9–16.7]	0.871	---	---	---	14.7	[12.9–16.7]	0.098
	Coupled	14.9	[13.5–16.4]		---	---	---	14.9	[13.5–16.4]	
	Not applicable	---	---	---	11.3	[9.1–13.9]	---	11.3	[9.1–13.9]	
**Main occupation during the past 12 months**	Trader/artisan	15.3	[13.5–17.5]	0.319	13.3	[3.3–53.1]	0.117	15.3	[13.5–17.4]	0.107
	Housewife/unemployed	14.1	[12.1–16.4]		11.9	[1.7–84.2]		14.1	[12.1–16.4]	
	Student	13.7	[10.9–17.2]		9.5	[6.9–13.0]		11.9	[9.9–14.3]	
	Other	15.7	[13.4–18.3]		---	---		15.6	[13.3–18.3]	
	Missing data	---	---	---	13.4	10.0–18.0		12.9	[9.6–17.3]	

Yo: years-old; CI: Confidence Interval

**Fig. 4:**
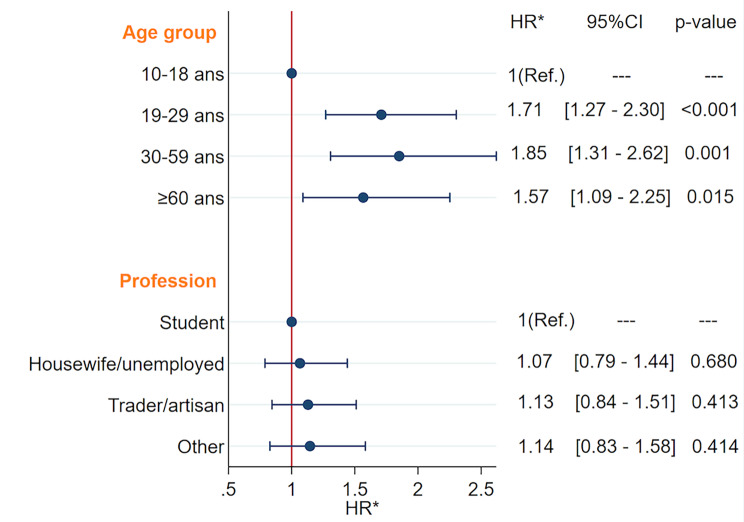
Predictors of SARS-CoV-2 seroconversion in the two largest cities of Burkina Faso (Ouagadougou and Bobo-Dioulasso) Yo: years-old; Others (retired = 102, employees = 80, others = 69) HR*: Hazard Ratio stratified by city (Cox proportional hazards regression)

Results of the bivariable analysis are reported in Table 3. The proportional hazards assumption was not verified for the variable “City of residence”. By multivariable analysis, only age group, adjusted for occupation (and stratified by city of residence), was predictive of seroconversion. The risk of seroconversion was almost 2 times higher in those aged 19 years and older than in those aged 10–18 years (HR = 1.7 [CI 1.3; 2.3], p < 0.001). Compared with participants aged 10–18 years, the risk of seroconversion was 1.7 [CI 1.3; 2.3], 1.9 [CI 1.3; 2.6], and 1.6 [CI 1.1; 2.3] times higher, respectively, in those aged 19–29 years, 30–39 years, and 60 years and over (Fig. 4).

**Table 3:** Predictors of SARS-COV-2 seroconversion (Cox proportional hazards regression, bivariable analysis)

		HR	95%CI	p-value
**Population group**				
≥ 60 yo (ref.)		---		
Males 19–59 yo		1.06	[0.87–1.29]	0.543
Female 19–29 yo		1.12	[0.93–1.35]	0.246
15–18 yo		0.64	[0.45–0.92]	0.015
10–14 yo		0.55	[0.40–0.76]	< 0.001
**Sex**				
Female (ref.)		---		
Male		1.01	[0.87–1.17]	0.898
**Age (years)**				
10–18 (ref.)		---		
19–29		1.79	[1.37–2.33]	< 0.001
30–59		1.91	[1.49–2.45]	< 0.001
≥ 60		1.70	[1.32–2.20]	< 0.001
**Adult**				
No (10–18 yo)		---		
Yes (≥ 19 yo)		1.80	[1.43–2.27]	< 0.001
**Educational level**				
Not enrolled in school (ref.)		---		
Literate/primary		0.89	[0.73–1.09]	0.262
Secondary		0.99	[0.82–1.20]	0.947
University		0.94	[0.70–1.24]	0.652
**Marital status**				
Single (ref.)		---		
Couple		1.01	[0.86–1.19]	0.924
Not applicable		0.56	[0.43–0.72]	< 0.001
**Main occupation during the past 12 months**				
Student (ref.)		---		
Housewife/unemployed		1.32	[1.05–1.65]	0.017
Trader/artisan		1.45	[1.18–1.79]	< 0.001
Other		1.47	[1.17–1.85]	0.001

h: Hazard Ratio, Yo: years-old, CI: Confidence Interval

Among those who seroconverted during the study (711/1399, 50.8%), 10–18 year-old participants (62/85, 72.9%) were more likely to be asymptomatic compared with participants aged 19 years and older (253/626, 40.4%) (p < 0.001).

## Discussion

Our study found an incidence rate of SARS-CoV-2 seroconversion of 14.3 cases per 100 person-weeks for pooled data from Ouagadougou and Bobo-Dioulasso. The cumulative incidence over our survey period was 50.8% (711/1399). Over the same period (March 3 to May 15, 2021), the health authorities of Burkina Faso reported 1348 new cases to WHO of COVID-19 occurring throughout the country [17]. Even if all these cases were reported for the cities of Ouagadougou and Bobo-Dioulasso alone, they would correspond to a cumulative incidence over the period of 0.0374% (1348/3,600,000), i.e. more than 1350 times (50.8/0.0374 = 1358) lower than the incidence reported in our study. This discrepancy could be explained by the difference in approaches. Indeed, the cases reported by the health authorities were most often symptomatic cases diagnosed by PCR in health facilities [18]. Routine screening was only done by PCR in contact cases and international travelers. This approach, although specific, is less sensitive in detecting SARS-CoV-2 infections than the systematic screening we performed by serological methods [19, 20]. In addition, with delays in consultations, the chances of isolating viral particles in the upper respiratory tract decrease, and thus the risk of having a negative PCR test increases. Serological test kits provide an estimate of the seroprevalence (past infections, and also current infections if the test kit includes anti-IgM), whereas PCR only diagnoses current acute infections. Another consideration is the reluctance of the population to be screened, even in the case of suspicious symptoms or proven contact with a confirmed case of COVID-19. This has been reported in some African countries such as Nigeria and South Africa, where a large proportion of the population did not believe in the existence of the disease or were very suspicious of the treatment offered [21, 22]. This reluctance of the population likely contributed to the diagnostic gap in the strategy implemented by the health authorities.

COVID-19 is essentially transmitted by the respiratory route and therefore the incidence could be higher in large cities because of the high population density [23–25]. This could explain the higher incidence observed in Ouagadougou compared to Bobo-Dioulasso. A cohort study of COVID-19 seroconversion in Ethiopia found incidence rates of 1622 cases [1004–2429] per 100,000 person-weeks and 4646 cases [2797–7255] per 100,000 person-weeks in Jimma and Addis Ababa, respectively [26]. These incidence rates are lower than those found in our cohort and may be related, in part, to the duration of the Ethiopian study, which lasted 8 months compared with 2 months for our study. In addition, there is a seasonality in the transmission of COVID-19 with peaks during cold periods [27, 28]. The Ethiopian study period has a long phase of low transmission (August-November 2020) and this may contribute to a lower overall incidence. Addis Ababa is a larger city than Jimma and the trend of higher incidence of COVID-19 in larger cities is also observed here.

Since the beginning of the COVID-19 pandemic, statistics reported by different countries indicate a low number of COVID-19 cases in children [29, 30]. Similarly, a meta-analysis of COVID-19 mortality from European, Asian, American, and Australian data indicated a lower mortality rate in children [31]. To explain this low severity (morbidity and mortality) of SARS-CoV-2 infection in children, the most common hypothesis is the low prevalence of comorbidities such as hypertension and diabetes in children [30]. In addition, in sub-Saharan Africa, co-infections and BCG vaccination are also mentioned, especially parasitic co-infections (helminthiasis, malaria). These co-infections, which are particularly frequent in children in this part of the world, are thought to have a stimulating effect on the immune system and make it more resistant to SARS-CoV-2 infection [32, 33]. Similarly, cross-immunity in children due to previous infections with other coronaviruses has also been suggested [30, 33]. While the available data tend to indicate a low number of COVID-19 illnesses and deaths in children, little information exists on seroconversion in children. In our study, the risk of seroconversion was twice as low in participants aged 10–18 years old than in those aged 19 years and over. This could be explained by the closure of classrooms and travel restrictions for children, orchestrated by the Burkinabe government and parents respectively during the pandemic. Indeed, these measures limited the chances of children being exposed to the virus. Almost three quarters (72.9%) of 10–18 year-olds with seroconversion reported no symptoms of COVID-19. This high proportion of asymptomatic COVID-19 infections in children has been reported in several studies [30, 34, 35]. The low morbidity of COVID-19 in children is likely multifactorial, involving limited exposure to the virus and under-screening/diagnosis because of the predominance of asymptomatic presentation.

### Limitations of study

Our study has some limitations. The first limitation is the high rate of loss to follow up among participants aged 19 years or over (31.8%). There were delays with the delivery of the test kits provided by WHO, and thus, we were unable to process the samples and get the results to participants before the next round of follow-up visits. Some participants were unwilling to continue the study and give a new sample before receiving the results of their previous tests. This high attrition rate could alter the true incidence rate of seroconversion, as well as the identification of predictive factors, if those lost to follow up differed fundamentally from participants who were followed up at all visits. However, we compared the characteristics of those lost to follow up with those of the full-term participants, and the characteristics were similar, apart from a slight predominance of men among those who dropped out (Table S3 in Additional file 2). The second limitation is that our study was carried out only in the two largest cities in Burkina Faso, which were the epicenter of the pandemic, and therefore the results are not applicable to the whole country. However, despite these limitations, this study is, to the best of our knowledge, the first on the incidence of seroconversion of SARS-CoV-2 infection in Burkina Faso and one of the few cohort studies on seroconversion to SARS-CoV-2 conducted in Africa. This enabled comparison with the data reported by the country’s health authorities and revealed the extent of the disease, contrary to the statistics reported by the national health authorities.

## Conclusion

This first study on the incidence of COVID-19 in Burkina Faso found an incidence rate in this study sample that was nearly 1350 times higher than that reported by the health authorities. It also indicates a higher incidence rate with the level of urbanization, and a very high proportion of asymptomatic cases, especially among children. The risk of seroconversion is twice as high in adults. This important information should be considered in the strategy to control COVID-19 in Burkina Faso and indeed in sub-Saharan Africa. Preventive measures such as physical distancing, wearing a mask, disinfecting or washing hands regularly and vaccination, should be more stringent in large cities. Adults living in large cities should be the priority targets for vaccination efforts against COVID-19. A national seroprevalence survey could provide an opportunity to assess the quantity and quality of anti-SARS-CoV-2 antibodies throughout the country, with a view to taking this into account in the national vaccination strategy against the disease.

## Electronic supplementary material

Below is the link to the electronic supplementary material.

Supplementary Material 1

Supplementary Material 2

Supplementary Material 3

Supplementary Material 4

## Data Availability

All data collected as part of this study will be made available upon reasonable request. Request should be addressed to the South-Side Principal investigator of the project Dr Isidore Tiandiogo Traoré (tiandiogo2002@yahoo.fr).

## Abbreviations

ANRS: France Recherche Nord & Sud Sida-hiv Hépatites
BCG: Bacille de Calmette et Guérin
CI: Confidence interval
COVID-19: Coronavirus disease 2019
EA: Enumeration area
ELISA: Enzyme-Linked Immuno Assay
EmulCOVID-19: Etude multidisciplinaire sur la COVID-19 (multidisciplinary research on COVID-19)
HZ: Hazard ratio
IgG: Immunoglobulin G
IgM: Immunoglobulin M
IRR: Incidence rate ratio (or rate ratio)
PCR: Polymerase chain reaction
SARS-CoV-2: Severe Acute Respiratory Syndrome Coronavirus 2
Yo: Years-old
WHO: World health organization
