# Supplementary material for: Incidence rate and predictors of COVID-19 in the two largest cities of Burkina Faso - prospective cohort study in 2021 (ANRS-COV13)
Source: BMC Infect Dis. 2023 Jun 12;23:394. doi: 10.1186/s12879-023-08361-2 (PMC10258776; doi:10.1186/s12879-023-08361-2)
Supplement: Supplementary file 1 — Supplementary Material 1 [file 12879_2023_8361_MOESM1_ESM.pdf]

QUESTIONNAIRE

Incidence rate and predictors of COVID-19 in the two largest cities of Burkina Faso - prospective cohort study in 2021 (ANRS-COV13)

Multidisciplinary Study of COVID-19 in Burkina Faso (EMuL-COVID-19), ANRS-COV13: sero- epidemiological survey

Inclusion Visit (D0)

ID Participant: City: |____| Household: |____|____|____| Participant: |____|____|____|

1. General Information

Date	└─┴─┘ └─┴─┘ └─┴─┴─┴─┘ Day/Month/Year	I1
Date of signature of informed consent	└─┴─┘ └─┴─┘ └─┴─┴─┴─┘ Day/Month/Year	I2
Field investigator name (last and first name)		I3

Study site	Ouagadougou 1<br>Bobo-Dioulasso 2	I4
Participant household identification number		I5
Participant identification number		I6
Participant phone number		I7

2. Socio-demographic Characteristics

**Question**	**Response**	**Code**
Sex?	Male 1 Female 2	Q001
Weight?	└─┴─┴─┘ . └─┘ Kg	Q002
Height?	└─┴─┴─┘ cm	Q003
How old are you??	Years └─┴─┘	Q004

Please indicate your marital status	Single 1<br>Married 2<br>Separated/divorced 3<br>Widowed 4<br>Living maritally 5<br>No response 77	Q006
Which of the following categories best describes your main professional activity over the last 12 months?	Civil servant (State) 1<br>Civil servant (Private) 2<br>Tradesman 3<br>Volunteer 4<br>Student 5<br>Homemaker/housewife 6<br>Informal other than trade 7<br>Retired 8<br>Jobseeker 9<br>Invalidity 10<br>Other 11<br>If other, specify: …………………<br>No response 77	Q007

ID Participant: City: |____| Household: |____|____|____| Participant: |____|____|____|

3. Context and medical history (chronic disease/immunosuppression)

Do you have any of the following diseases?

**Question**	**Response**	**Code**
Diabetes?	Yes 1<br>No 2<br>Don't know/ no answer 77<br>If yes, specify the type (1 or 2) if known: ………..	Q008
Arterial hypertension?	Yes 1<br>No 2<br>Don't know/ no answer 77	Q009
Chronic renal disease?	Yes 1<br>No 2<br>Don't know/ no answer 77<br><br>If yes, specify: …………………………………………….	Q010
Chronic liver disease?	Yes 1<br>No 2<br>Don't know/ no answer 77<br><br>If yes, specify: …………………………………………….	Q011
Chronic heart disease?	Yes 1<br>No 2<br>Don't know/ no answer 77<br>If yes, specify: …………………………………………….	Q012
Chronic neurological or neuromuscular disease?	Yes 1<br>No 2<br>Don't know/ no answer 77<br><br>If yes, specify: …………………………………………….	Q012a
Chronic rheumatological disease?	Yes 1<br>No 2<br>Don't know/ no answer 77<br><br>If yes, specify: …………………………………………….	Q012b
HIV infection?	Yes 1<br>No 2<br>Don't know/ no answer 77	Q013
Pulmonary tuberculosis?	Yes 1<br>No 2<br>Don't know/ no answer 77	Q014
Other chronic respiratory diseases (asthma or COPD*)?	Yes 1<br>No 2<br>Don't know/ no answer 77<br><br>if yes specify :	Q015

***COPD**: chronic obstructive pulmonary disease

ID Participant: City: |____| Household: |____|____|____| Participant: |____|____|____|

Pregnancy?	Yes 1<br>No 2<br>Don't know/ no answer / Not applicable 77<br>If yes, gestational age (weeks of amenorrhea) :	Q016
History of BCG vaccination documented/self-reported?	Yes (vaccination record) 1<br>Yes (injection site scar) 2<br>Yes (self-reported) 3<br>No 4	Q017
Malignant tumor?	Yes 1<br>No 2<br>Don't know/ no answer 77<br>if yes specify:	Q018
Other chronic disease (specify)?<br>……………………………………………	Yes 1<br>No 2<br>Don't know/ no answer 77	Q019
Have you ever received a vaccine against COVID-19?	Yes (vaccination record) 1<br>Yes (injection site scar) 2<br>Yes (self-reported) 3<br>Specify if other document than the vaccination record<br>No (if no, go to question Q020) 4<br>Other 5<br>If other, specify: …………………………………	Q019a
If yes,		
Specify if other document than the vaccination record		Q019b
Specify the country		Q019c
Number of doses received?	1 or 2	Q019d
Origin of the vaccine received?<br>*Many possible answers*	Self-reported or reported by a relative 1<br>Vaccination card 2<br>Immunization Registry 3<br>Medical file 4<br>Other 5<br>If other, specify: ………………………………..	Q019e
Date of the 1st dose?<br>Name of the vaccine:	|__|__| |__|__| |__|__|__|__| Day/Month/Year<br>………………………………………………………………………	Q019f<br>Q019g
Date of the 2nd dose?<br>Name of the vaccine :	|__|__| |__|__| |__|__|__|__| Day/Month/Year<br>………………………………………………………………………	Q019h<br>Q019i

4. Lifestyle and ongoing treatment

Question	Response	Code
Current smoker?	Yes 1<br>No 2<br>Don't know/ no answer NK	Q020
Former smoker?	Yes 1<br>No 2<br>Don't know/ no answer NK	Q021
If current or former smoker, duration of smoking?	└─┴─┘. └─┘ years	Q022
How many cigarettes do (did) you smoker per day?	└─┴─┴─┘ cigarettes per day	Q023
Within the past 12 months, how often did you have a drink containing alcohol?	Never 0<br>Once a month or less 1<br>2 to 4 times per month 2<br>2 to 3 times per week 3<br>At least 4 times per week 4<br>Don't know/ no answer NK	Q024<br>***If 0, go to Q028***
How many drinks containing alcohol did you have on a typical day when you were drinking?	1 or 2 0<br>3 or 4 1<br>5 or 6 2<br>7 to 9 3<br>10 or more 4<br>Don't know/ no answer NK	Q025
How often did you have six or more drinks on one occasion	Never 0<br>Once a month or less 1<br>2 to 4 times per month 2<br>2 to 3 times per week 3<br>At least 4 times per week 4<br>Don't know/ no answer NK	Q026
AUDIT Score, short version (sum of the scores for the 3 previous items)	└─┴─┘	Q027
Long term corticosteroid therapy (> 10 days of treatment)?	Yes 1<br>No 2<br>Don't know/ no answer 77	Q028
Immunosuppressive therapy (radiotherapy or chemotherapy)?	Yes 1<br>No 2<br>Don't know/ no answer 77	Q029
Ongoing antiretroviral therapy?	Yes 1<br>No 2<br>Don't know/ no answer 77	Q030
Patient on dialysis?	Yes 1<br>No 2<br>Don't know/ no answer 77	Q031

ID Participant: City: |____| Household: |____|____|____| Participant: |____|____|____|

5. Characterization of the exposure to COVID-19

Question	Response	Code
Have you ever had a nasopharyngeal swab for COVID-19?	Yes 1<br>No 2 If not go to the question Q036<br>Don't know/ no answer 77	Q032
if yes,		
Date?	|__|__| |__|__| |__|__|__|__| Day/Month/Year	Q033
Why?	Suspect case 1<br>Contact case 2<br>Border / airport control 3<br>Voluntary testing 4<br>Don't know / no answer 77	Q034
What was the result?	Positive 1<br>Negative 2<br>Don't know/ no answer 77	Q035
Self-medication (preventive or curative) against COVID-19?	Yes 1<br>No 2<br>Don't know/ no answer 77	Q036
If yes,		
Indicate the treatment taken	Chloroquine/hydroxychloroquine 1<br>Chloroquine/hydroxychloroquine + AZ* 2<br>Traditional herbal medicine 3<br>Other treatment (specify) 4<br>………………………………………………………	Q037<br>***if 1,2 or 4, go to Q038***

***AZ**: Azithromycin

ID Participant: City: |____| Household: |____|____|____| Participant: |____|____|____|

USERS OF MEDICINAL PLANTS

**Question**	**Response**	**Code**
Was the herbal medicine prescribed by	Traditional practitioner 1<br>Grandma's Recipe 2<br>Other 3<br>If other, specify………………………………………..	Q037a

Which plant (s) do you use or did you use for the treatment of covid-19?	Name of the plants		**Parts of the plant used**	Q037b
	In local languages	Correspondence in French		

Can you describe the preparation of the herbal medicine? (Carefully note all the steps and their duration)	…………………………………………………………….<br>……………………………………………………………<br>……………………………………………………………<br>……………………………………………………………<br>……………………………………………………………<br>…………………………………………………………….<br>…………………………………………………………….<br>…………………………………………………………….<br>…………………………………………………………….<br>…………………………………………………………….<br>…………………………………………………………….	Q037c

ID Participant: City: |____| Household: |____|____|____| Participant: |____|____|____|

What is the form of the phytomedicine at the time of its use?	Infusion 1<br>Decoction 2<br>Maceration 3<br>Smoke 4<br>Steam 5<br>Other 6<br>If other, specify………………………………………..	Q037d
How is the phytomedicine used?	Cold drink 1<br>Hot drink 2<br>Body bath 3<br>Inhalation / Fumigation 4<br>Application / Massage 5<br>Enema (purge) 6<br>Other mode of use 7<br>If other, specify ………………………………………..	
How long does the treatment last?	1 to 3 days 1<br>4 to 7 days 2<br>1 week to 1 month 3<br>1 to 3 months 4<br>Several years 5	Q037f
What is the dosage of the treatment?<br>Where do you find the treatment?<br>How did you use it?	……………………………………………………………….<br>………………………………………………………………<br>……………………………………………………………<br>……………………………………………………………….<br>………………………………………………………………<br>……………………………………………………………<br>……………………………………………………………….<br>………………………………………………………………<br>……………………………………………………………	Q037g
Are there any prohibitions during the treatment?	Yes 1<br>No 2<br>Don't know/ no answer 77	Q037h
What are the prohibitions during the treatment?	……………………………………………………………….<br>………………………………………………………………<br>………………………………………………………………<br>………………………………………………………………	Q037i

ID Participant: City: |____| Household: |____|____|____| Participant: |____|____|____|

Did you in the last 14 days:		
**Question**	**Response**	**Code**
Visit COVID-19 patients?	Yes 1<br>No 2<br>Don't know/ no answer 77	Q038
Work with people with COVID-19?	Yes 1<br>No 2<br>Don't know/ no answer 77	Q039
Have a face-to-face contact with a COVID-19 patient at a distance of less than 1m?	Yes 1<br>No 2<br>Don't know/ no answer 77	Q040
Shared the same enclosed environment as a COVID-19 patient (including sharing a classroom or housekeeping or being at the same gathering)?	Yes 1<br>No 2<br>Don't know/ no answer 77	Q041
Traveled (coach / bus / taxi / personal car / plane) with a COVID-19 patient?	Yes 1<br>No 2<br>Don't know/ no answer 77	Q042
Provided direct care to COVID-19 patients?	Yes 1<br>No 2<br>Don't know/ no answer 77	Q043

ID Participant: City: |____| Household : |____|____|____| Participant : |____|____|____|

6. Clinical Characteristics

1st confirmed case of COVID-19 in Burkina Faso: 03/09/2020
During the past 14 days have you experienced / exhibited any of the following clinical signs:

**Question**	**Response**	**Code**
Fever (≥38 ° C) or history of fever?	Yes 1<br>No 2<br>Don't know/ no answer 77	Q044
Asthenia / fatigue / malaise?	Yes 1<br>No 2<br>Don't know/ no answer 77	Q045
Myalgia / stiffness?	Yes 1<br>No 2<br>Don't know/ no answer 77	Q046
Joint pain?	Yes 1<br>No 2<br>Don't know/ no answer 77	Q047
Headache?	Yes 1<br>No 2<br>Don't know/ no answer 77	Q048
Chills (feeling cold)?	Yes 1<br>No 2<br>Don't know/ no answer 77	Q049
Cough?	Yes 1<br>No 2<br>Don't know/ no answer 77	Q050
Dyspnea (difficulty in breathing)?	Yes 1<br>No 2<br>Don't know/ no answer 77	Q051
Wheezing	Yes 1<br>No 2<br>Don't know/ no answer 77	Q052
Cold?	Yes 1<br>No 2<br>Don't know/ no answer 77	Q053
Epistaxis (nosebleed)?	Yes 1<br>No 2<br>Don't know/ no answer 77	Q054
Sore throat / angina?	Yes 1<br>No 2<br>Don't know/ no answer 77	Q055
Nausea / Vomiting?	Yes 1<br>No 2<br>Don't know/ no answer 77	Q056
Abdominal pain?	Yes 1<br>No 2<br>Don't know/ no answer 77	Q057

ID Participant: City: |____| Household: |____|____|____| Participant: |____|____|____|

Diarrhea?	Yes 1<br>No 2<br>Don't know/ no answer 77	Q058
Conjunctivitis?	Yes 1<br>No 2<br>Don't know/ no answer 77	Q059
Ageusia (loss of taste)?	Yes 1<br>No 2<br>Don't know/ no answer 77	Q060
Anosmia (loss of smell)?	Yes 1<br>No 2<br>Don't know/ no answer 77	Q061
Anorexia (loss of appetite)?	Yes 1<br>No 2<br>Don't know/ no answer 77	Q062
Rash (rashes)?	Yes 1<br>No 2<br>Don't know/ no answer 77	Q063
Disorders of consciousness?	Yes 1<br>No 2<br>Don't know/ no answer 77	Q064
Convulsions?	Yes 1<br>No 2<br>Don't know/ no answer 77	Q065
Other symptoms (to be specified)?<br>……………………………………………	Yes 1<br>No 2<br>Don't know/ no answer 77	Q066

Complications		
**Question**	**Response**	**Code**
Did you consult because of these symptoms / signs?	Yes 1<br>No 2<br>Don't know/ no answer 77	Q067
Have these symptoms / signs caused you to miss school or work?	Yes 1<br>No 2<br>Don't know/ no answer 77	Q068
Did these symptoms / signs require hospitalization?	Yes 1<br>No 2<br>Don't know/ no answer 77	Q069

ID Participant: City: |____| Household: |____|____|____| Participant: |____|____|____|

7. Venous blood collection		
**Question**	**Response**	**Code**
Blood sample taken?	Yes 1<br>No 2<br>If no, Why? ……………………………………..<br><br>………………………………………………………………	Q070
Time of sampling?	└─┴─┘ hours └─┴─┘ minutes	Q070a
DBS realized?	Yes 1<br>No 2	Q071
Incident occurred during the sampling?	Yes 1<br>No 2<br>If yes, give details: ……………………………………..<br>………………………………………………………………	Q072

Name and surname of the field investigator Date and signature

ID Participant: City: |____| Household: |____|____|____| Participant: |____|____|____|

8. D0 laboratory results

Methods and results of serological tests

**Question**	**Response**	**Code**
Sample number?	└─┴─┴─┴─┴─┘	Q073
Sampling date?	└─┴─┘ └─┴─┘ └─┴─┴─┴─┘<br>Day/Month/Year	Q074
Time of sampling?	└─┴─┘ hours └─┴─┘ minutes	Q075
Date received at the laboratory?	└─┴─┘ └─┴─┘ └─┴─┴─┴─┘<br>Day/Month/Year	Q076
Reception time at the laboratory?	└─┴─┘ hours └─┴─┘ minutes	Q077
Type of sample?	Serum 1<br>Other type (specify) 2<br>……………………………………………………………	Q078
Result of the serological test?	IgG ☐ Positive ☐ Negative ☐ Undetermined<br>IgM ☐ Positive ☐ Negative ☐ Undetermined	Q079
Result of the viral load?		Q080
Sample transferred to another laboratory for confirmation?	Yes 1<br>No 2	Q081
if Yes,		
Name and address of the laboratory for confirmation		Q082
When was the sample sent?	└─┴─┘ └─┴─┘ └─┴─┴─┴─┘<br>Day/Month/Year	Q083
When was the result confirmed?	└─┴─┘ └─┴─┘ └─┴─┴─┴─┘<br>Day/Month/Year	Q084
What is the serological test confirmation result?	IgG ☐ Positive ☐ Negative ☐ Undetermined<br>IgM ☐ Positive ☐ Negative ☐ Undetermined	Q085

Name and surname of the biologist Date and signature

ID Participant : City: |____| Household: |____|____|____| Participant: |____|____|____|

Name and surname of the participant	

Multidisciplinary Study of COVID-19 in Burkina Faso (EMuL-COVID-19), ANRS-COV13: sero- epidemiological survey

Follow-up visit (D21)

1. Characterization of the exposure to COVID-19

**Question**	**Response**	**Code**
Have you ever had a nasopharyngeal swab for COVID-19?	Yes 1<br>No 2 If not, go to the question Q090<br>Don't know/ no answer 77	Q086
If yes,		
Date?	|__|__| |__|__| |__|__|__|__| Day/Month/Year	Q087
Why?	Suspect case 1<br>Contact case 2<br>Border / airport control 3<br>Don't know/ no answer 77	Q088
What was the result?	Positive 1<br>Negative 2<br>Don't know/ no answer 77	Q089
Self-medication (preventive or curative) against COVID-19?	Yes 1<br>No 2<br>Don't know/ no answer 77	Q090
If yes,		
Indicate the treatment taken	Chloroquine/hydroxychloroquine 1<br>Chloroquine/hydroxychloroquine + AZ* 2<br>Traditional herbal medicine 3<br>Other treatment (specify) 4<br>………………………………………………………………	Q091<br>***if 1,2 or 4, go to Q092***

***AZ**: Azithromycin

ID Participant: City: |____| Household: |____|____|____| Participant: |____|____|____|

USERS OF MEDICINAL PLANTS

Question	Response	Code
**Was the herbal medicine prescribed by**	Traditional practitioner 1<br>Grandma's Recipe 2<br>Other 3<br>If other, specify………………………………………..	Q091a

Which plant (s) do you use or did you use for the treatment of covid-19?	**Name of the plants**		**Parts of the plant used**	Q091b
	In local languages	Correspondence in French		

Can you describe the preparation of the herbal medicine? (Carefully note all the steps and their duration)	……………………………………………………………….<br>………………………………………………………………<br>………………………………………………………………<br>………………………………………………………………<br>………………………………………………………………<br>……………………………………………………………….<br>……………………………………………………………….<br>……………………………………………………………….<br>……………………………………………………………….<br>……………………………………………………………….<br>……………………………………………………………….	Q091c

ID Participant: City: |____| Household: |____|____|____| Participant: |____|____|____|

What is the form of the phytomedicine at the time of its use?	Infusion 1<br>Decoction 2<br>Maceration 3<br>Smoke 4<br>Steam 5<br>Other 6<br>If other, specify………………………………………..	Q091d
How is the phytomedicine used?	Cold drink 1<br>Hot drink 2<br>Body bath 3<br>Inhalation / Fumigation 4<br>Application/Massage 5<br>Enema (purge) 6<br>Other mode of use 7<br>If other, specify………………………………………..	Q091e
How long does the treatment last?	1 to 3 days 1<br>4 to 7 days 2<br>1 week to 1 month 3<br>1 to 3 months 4<br>Several years 5	Q091f
What is the dosage of the treatment?<br><br>How did you use it?	……………………………………………………………….<br>………………………………………………………………<br>………………………………………………………………<br>………………………………………………………………<br>………………………………………………………………<br>……………………………………………………………….<br>……………………………………………………………….<br>………………………………………………………………<br>……………………………………………………………….<br>……………………………………………………………….	Q091g

ID Participant: City: |____| Household: |____|____|____| Participant: |____|____|____|

Are there any prohibitions during the treatment?	Yes 1<br>No 2<br>Don't know/ no answer 77	Q091h
What are the prohibitions during the treatment?	……………………………………………………………….<br>………………………………………………………………<br>………………………………………………………………<br>………………………………………………………………	Q091i

Did you in the last 21 days (Since the last visit):		
**Question**	**Response**	**Code**
Visit COVID-19 patients?	Yes 1<br>No 2<br>Don't know/ no answer 77	Q092
Work with people with COVID-19?	Yes 1<br>No 2<br>Don't know/ no answer 77	Q093
Have a face-to-face contact with a COVID-19 patient at a distance of less than 1m?	Yes 1<br>No 2<br>Don't know/ no answer 77	Q094
Shared the same enclosed environment as a COVID-19 patient (including sharing a classroom or housekeeping or being at the same gathering)?	Yes 1<br>No 2<br>Don't know/ no answer 77	Q095
Traveled (coach / bus / taxi / personal car / plane) with a COVID-19 patient?	Yes 1<br>No 2<br>Don't know/ no answer 77	Q096
Provided direct care to COVID-19 patients?	Yes 1<br>No 2<br>Don't know/ no answer 77	Q097

ID Participant: City: |____| Household: |____|____|____| Participant: |____|____|____|

2. Clinical Characteristics

1st confirmed case of COVID-19 in Burkina Faso: 03/09/2020
During the past 21 days have you experienced / exhibited any of the following clinical signs

**Question**	**Response**	**Code**
Fever (≥38 ° C) or history of fever?	Yes 1<br>No 2<br>Don't know/ no answer 77	Q098
Asthenia / fatigue / malaise?	Yes 1<br>No 2<br>Don't know/ no answer 77	Q099
Myalgia / stiffness?	Yes 1<br>No 2<br>Don't know/ no answer 77	Q100
Joint pain?	Yes 1<br>No 2<br>Don't know/ no answer 77	Q101
Headache?	Yes 1<br>No 2<br>Don't know/ no answer 77	Q102
Chills (feeling cold)?	Yes 1<br>No 2<br>Don't know/ no answer 77	Q103
Cough?	Yes 1<br>No 2<br>Don't know/ no answer 77	Q104
Dyspnea (difficulty in breathing)?	Yes 1<br>No 2<br>Don't know/ no answer 77	Q105
Wheezing	Yes 1<br>No 2<br>Don't know/ no answer 77	Q106
Cold?	Yes 1<br>No 2<br>Don't know/ no answer 77	Q107
Epistaxis (nosebleed)?	Yes 1<br>No 2<br>Don't know/ no answer 77	Q108
Sore throat / angina?	Yes 1<br>No 2<br>Don't know/ no answer 77	Q109
Nausea / Vomiting?	Yes 1<br>No 2<br>Don't know/ no answer 77	Q110

ID Participant: City: |____| Household: |____|____|____| Participant: |____|____|____|

Abdominal pain?	Yes 1<br>No 2<br>Don't know/ no answer 77	Q111
Diarrhea?	Yes 1<br>No 2<br>Don't know/ no answer 77	Q112
Conjunctivitis?	Yes 1<br>No 2<br>Don't know/ no answer 77	Q113
Ageusia (loss of taste)?	Yes 1<br>No 2<br>Don't know/ no answer 77	Q114
Anosmia (loss of smell)?	Yes 1<br>No 2<br>Don't know/ no answer 77	Q115
Anorexia (loss of appetite)?	Yes 1<br>No 2<br>Don't know/ no answer 77	Q116
Rash (rashes)?	Yes 1<br>No 2<br>Don't know/ no answer 77	Q117
Disorders of consciousness?	Yes 1<br>No 2<br>Don't know/ no answer 77	Q118
Convulsions?	Yes 1<br>No 2<br>Don't know/ no answer 77	Q119
Other symptoms (to be specified)?<br>……………………………………………	Yes 1<br>No 2<br>Don't know/ no answer 77	Q120
Did you consult because of these symptoms / signs?	Yes 1<br>No 2<br>Don't know/ no answer 77	Q121
Have these symptoms / signs caused you to miss school or work?	Yes 1<br>No 2<br>Don't know/ no answer 77	Q122
Did these symptoms / signs require hospitalization?	Yes 1<br>No 2<br>Don't know/ no answer 77	Q123

ID Participant: City: |____| Household: |____|____|____| Participant: |____|____|____|

3. Venous blood collection		
**Question**	**Response**	**Code**
Blood sample taken?	Yes 1<br>No 2<br>If no, Why? ……………………………………..<br>………………………………………………………………	Q124
Time of sampling?	└─┴─┘ hours └─┴─┘ minutes	Q124a
DBS realized?	Yes 1<br>No 2	Q125
Incident occurred during the sampling?	Yes 1<br>No 2<br>If yes, give details: ……………………………………..<br>………………………………………………………………	Q126

Name and surname of the field investigator Date and signature

ID Participant: City: |____| Household: |____|____|____| Participant: |____|____|____|

4. laboratory results J21		
**Méthodes and résultats des tests sérologiques**		
**Question**	**Response**	**Code**
Sample number?	└─┴─┴─┴─┴─┘	Q127
Sampling date?	└─┴─┘ └─┴─┘ └─┴─┴─┴─┘<br>Day/Month/Year	Q128
Time of sampling?	└─┴─┘ hours └─┴─┘ minutes	Q129
Date received at the laboratory?	└─┴─┘ └─┴─┘ └─┴─┴─┴─┘<br>Day/Month/Year	Q130
Reception time at the laboratory?	└─┴─┘ hours └─┴─┘ minutes	Q131
Type of sample?	Serum 1<br>Other type (specify) 2<br>………………………………………………………	Q132
Result of the serological test?	IgG ☐ Positive<br>☐ Negative<br>☐ Undetermined<br>IgM ☐ Positive<br>☐ Negative<br>☐ Undetermined	Q133
Result of the viral load?		Q134
Sample transferred to another laboratory for confirmation?	Yes 1<br>No 2	Q135
If yes,		
Name and address of the laboratory for confirmation		Q136
When was the sample sent?	└─┴─┘ └─┴─┘ └─┴─┴─┴─┘<br>Day/Month/Year	Q137
When was the result confirmed?	└─┴─┘ └─┴─┘ └─┴─┴─┴─┘<br>Day/Month/Year	Q138
What is the serological test confirmation result?	IgG ☐ Positive<br>☐ Negative<br>☐ Undetermined<br>IgM ☐ Positive<br>☐ Negative<br>☐ Undetermined	Q139

Name and surname of the biologist Date and signature

ID Participant: City: |____| Household: |____|____|____| Participant: |____|____|____|

Name and surname of the participant	

Multidisciplinary Study of COVID-19 in Burkina Faso (EMuL-COVID-19), ANRS-COV13: sero- epidemiological survey

Follow-up visit (D42)

1. Characterization of the exposure to COVID-19		
**Question**	**Response**	**Code**
Have you ever had a nasopharyngeal swab for COVID-19?	Yes 1<br>No 2 If not, go to the question Q144<br>Don't know/ no answer 77	Q140
If yes,		
Date?	|__|__| |__|__| |__|__|__|__| Day/Month/Year	Q141
Why?	Suspect case 1<br>Contact case 2<br>Border / airport control 3<br>Don't know/ no answer 77	Q142
What was the result?	Positive 1<br>Negative 2<br>Don't know/ no answer 77	Q143
Self-medication (preventive or curative) against COVID-19?	Yes 1<br>No 2<br>Don't know/ no answer 77	Q144
if yes,		
Indicate the treatment taken	Chloroquine/hydroxychloroquine 1<br>Chloroquine/hydroxychloroquine + AZ* 2<br>Traditional herbal medicine 3<br>Other treatment (specify) 4	Q145<br>***if 1,2 or 4, go to Q146***

ID Participant: City: |____| Household : |____|____|____| Participant : |____|____|____|

USERS OF MEDICINAL PLANTS

Question	Response	Code
**Was the herbal medicine prescribed by**	Traditional practitioner 1<br>Grandma's Recipe 2<br>Other 3<br>If other, specify………………………………………..	Q145a

Which plant (s) do you use or did you use for the treatment of covid-19?	**Name of the plants**		**Parts of the plant used**	Q145b
	In local languages	Correspondence in French		

Can you describe the preparation of the herbal medicine? (carefully note all the steps and their duration)	……………………………………………………………….<br>………………………………………………………………<br>………………………………………………………………<br>………………………………………………………………<br>………………………………………………………………<br>……………………………………………………………….<br>……………………………………………………………….<br>……………………………………………………………….<br>……………………………………………………………….<br>……………………………………………………………….	Q145c

ID Participant: City: |____| Household: |____|____|____| Participant : |____|____|____|

What is the form of the phytomedicine at the time of its use?	Infusion 1<br>Decoction 2<br>Maceration 3<br>Smoke 4<br>Steam 5<br>Other 6<br>If other, specify………………………………………..	Q145d
How is the phytomedicine used?	Cold drink 1<br>Hot drink 2<br>Body bath 3<br>Inhalation / Fumigation 4<br>Application/Massage 5<br>Enema (purge) 6<br>Other mode of use 7<br>If other, specify………………………………………..	Q145e
How long does the treatment last?	1 to 3 days 1<br>4 to 7 days 2<br>1 week to 1 month 3<br>1 to 3 months 4<br>Several years 5	Q145f
What is the dosage of the treatment?<br>What is the dosage of the treatment?<br>How did you use it?	……………………………………………………………….<br>………………………………………………………………<br>………………………………………………………………<br>………………………………………………………………<br>………………………………………………………………<br>……………………………………………………………….<br>……………………………………………………………….<br>……………………………………………………………….	Q145g

Are there any prohibitions during the treatment?	Yes 1<br>No 2<br>Don’t know/ no answer 77	Q145h
What are the prohibitions during the treatment?	…………………………………………………………….<br>……………………………………………………………<br>……………………………………………………………<br>……………………………………………………………	Q145i

Did you in the last 21 days:		
**Question**	**Response**	**Code**
Visit COVID-19 patients?	Yes 1<br>No 2<br>Don’t know/ no answer 77	Q146
Work with people with COVID-19?	Yes 1<br>No 2<br>Don’t know/ no answer 77	Q147
Have a face-to-face contact with a COVID-19 patient at a distance of less than 1m?	Yes 1<br>No 2<br>Don’t know/ no answer 77	Q148
Shared the same enclosed environment as a COVID-19 patient (including sharing a classroom or housekeeping or being at the same gathering)?	Yes 1<br>No 2<br>Don’t know/ no answer 77	Q149
Traveled (coach / bus / taxi / personal car / plane) with a COVID-19 patient?	Yes 1<br>No 2<br>Don’t know/ no answer 77	Q150
Provided direct care to COVID-19 patients?	Yes 1<br>No 2<br>Don’t know/ no answer 77	Q151

ID Participant: City: |____| Household: |____|____|____| Participant : |____|____|____|

2. Clinical Characteristics

1st confirmed case of COVID-19 in Burkina Faso: 03/09/2020
During the past 21 days have you experienced / exhibited any of the following clinical sign

Question	Response	Code
Fever (≥38 ° C) or history of fever?	Yes 1<br>No 2<br>Don't know/ no answer 77	Q152
Asthenia / fatigue / malaise?	Yes 1<br>No 2<br>Don't know/ no answer 77	Q153
Myalgia / stiffness?	Yes 1<br>No 2<br>Don't know/ no answer 77	Q154
Joint pain?	Yes 1<br>No 2<br>Don't know/ no answer 77	Q155
Headache?	Yes 1<br>No 2<br>Don't know/ no answer 77	Q156
Chills (feeling cold)?	Yes 1<br>No 2<br>Don't know/ no answer 77	Q157
Cough?	Yes 1<br>No 2<br>Don't know/ no answer 77	Q158
Dyspnea (difficulty in breathing)?	Yes 1<br>No 2<br>Don't know/ no answer 77	Q159
Wheezing	Yes 1<br>No 2<br>Don't know/ no answer 77	Q160
Cold?	Yes 1<br>No 2<br>Don't know/ no answer 77	Q161
Epistaxis (nosebleed)?	Yes 1<br>No 2<br>Don't know/ no answer 77	Q162
Sore throat / angina?	Yes 1<br>No 2<br>Don't know/ no answer 77	Q163
Nausea / Vomiting?	Yes 1<br>No 2<br>Don't know/ no answer 77	Q164

ID Participant: City: |____| Household: |____|____|____| Participant: |____|____|____|

Abdominal pain?	Yes 1<br>No 2<br>Don't know/ no answer 77	Q165
Diarrhea?	Yes 1<br>No 2<br>Don't know/ no answer 77	Q166
Conjunctivitis?	Yes 1<br>No 2<br>Don't know/ no answer 77	Q167
Ageusia (loss of taste)?	Yes 1<br>No 2<br>Don't know/ no answer 77	Q168
Anosmia (loss of smell)?	Yes 1<br>No 2<br>Don't know/ no answer 77	Q169
Anorexia (loss of appetite)?	Yes 1<br>No 2<br>Don't know/ no answer 77	Q170
Rash (rashes)?	Yes 1<br>No 2<br>Don't know/ no answer 77	Q171
Disorders of consciousness?	Yes 1<br>No 2<br>Don't know/ no answer 77	Q172
Convulsions?	Yes 1<br>No 2<br>Don't know/ no answer 77	Q173
Other symptoms (to be specified)?<br>…………………………………………	Yes 1<br>No 2<br>Don't know/ no answer 77	Q174
Did you consult because of these symptoms / signs?	Yes 1<br>No 2<br>Don't know/ no answer 77	Q175
Have these symptoms / signs caused you to miss school or work?	Yes 1<br>No 2<br>Don't know/ no answer 77	Q176
Did these symptoms / signs require hospitalization?	Yes 1<br>No 2<br>Don't know/ no answer 77	Q177

ID Participant: City: |____| Household: |____|____|____| Participant: |____|____|____|

3. Venous blood collection		
**Question**	**Response**	**Code**
Blood sample taken?	Yes 1<br>No 2<br>If no, Why? ……………………………………..<br>………………………………………………………………	Q178
Time of sampling?	└─┴─┘ hours └─┴─┘ minutes	Q178a
DBS realized?	Yes 1<br>No 2	Q179
Incident occurred during the sampling?	Yes 1<br>No 2<br>If yes, give details: ……………………………………..<br>………………………………………………………………	Q180

Name and surname of the field investigator Date and signature

ID Participant: City: |____| Household: |____|____|____| Participant: |____|____|____|

4. D42 laboratory results		
**Methods and results of serological tests (a new table for each sample collected)**		
**Question**	**Response**	**Code**
Sample number?		Q181
Sampling date?	|__|__| |__|__| |__|__|__|__| Day/Month/Year	Q182
Time of sampling?	|__|__| hours |__|__| minutes	Q183
Date received at the laboratory?	|__|__| |__|__| |__|__|__|__| Day/Month/Year	Q184
Reception time at the laboratory?	|__|__| hours |__|__| minutes	Q185
Type of sample?	Serum 1<br>Other type (specify) 2<br>…………………………………………………………	Q186
Result of the serological test?	IgG ☐ Positive ☐ Negative ☐ Undetermined<br>IgM ☐ Positive ☐ Negative ☐ Undetermined	Q187
Result of the viral load?		Q188
Sample transferred to another laboratory for confirmation?	Yes 1<br>No 2	Q189
if Yes,		
Name and address of the laboratory for confirmation		Q190
When was the sample sent?	|__|__| |__|__| |__|__|__|__| Day/Month/Year	Q191
When was the result confirmed?	|__|__| |__|__| |__|__|__|__| Day/Month/Year	Q192
What is the serological test confirmation result?	IgG ☐ Positive ☐ Negative ☐ Undetermined<br>IgM ☐ Positive ☐ Negative ☐ Undetermined	Q193

ID Participant : City: |____| Household: |____|____|____| Participant: |____|____|____|

Name and surname of the participant	

Multidisciplinary Study of COVID-19 in Burkina Faso (EMuL-COVID-19), ANRS-COV13: sero- epidemiological survey

Follow-up visit (D63)

1. Characterization of the exposure to COVID-19

Question	Response	Code
Have you ever had a nasopharyngeal swab for COVID-19?	Yes 1<br>No 2 If no, go to the question Q198<br>Don't know/ no answer 77	Q194
If yes,		
Date?	└─┴─┘ └─┴─┘ └─┴─┴─┴─┘<br>Day/Month/Year	Q195
Why?	Suspect case 1<br>Contact case 2<br>Border / airport control 3<br>Don't know/ no answer 77	Q196
What was the result?	Positive 1<br>Negative 2<br>Don't know/ no answer 77	Q197
Self-medication (preventive or curative) against COVID-19?	Yes 1<br>No 2<br>Don't know/ no answer 77	Q198
If yes,		
Indicate the treatment taken	Chloroquine/hydroxychloroquine 1<br>Chloroquine/hydroxychloroquine + AZ* 2<br>Traditional herbal medicine 3<br>Other treatment (specify) 4<br>………………………………………………………………	Q199<br>***if 1,2 or 4, go to Q200***

***AZ**: Azithromycin

ID Participant: City: |____| Household: |____|____|____| Participant: |____|____|____|

USERS OF MEDICINAL PLANTS		
**Question**	**Response**	**Code**
**Was the herbal medicine prescribed by**	Traditional practitioner 1<br>Grandma's Recipe 2<br>Other 3<br>If other, specify………………………………………..	Q199a

Which plant (s) do you use, or did you use for the treatment of covid-19?	**Name of the plants**		**Parts of the plant used**	Q199b
	In local languages	Correspondence in French		

Can you describe the preparation of the herbal medicine? (carefully note all the steps and their duration)	……………………………………………………………….<br>………………………………………………………………<br>………………………………………………………………<br>………………………………………………………………<br>………………………………………………………………<br>……………………………………………………………….<br>……………………………………………………………….<br>……………………………………………………………….<br>……………………………………………………………….<br>……………………………………………………………….<br>……………………………………………………………….	Q199c

ID Participant: City: |____| Household: |____|____|____| Participant: |____|____|____|

What is the form of the phytomedicine at the time of its use?	Infusion 1<br>Decoction 2<br>Maceration 3<br>Smoke 4<br>Steam 5<br>Other 6<br>If other, specify………………………………………..	Q199d
How is the phytomedicine used?	Cold drink 1<br>Hot drink 2<br>Body bath 3<br>Inhalation / Fumigation 4<br>Application/Massage 5<br>Enema (purge) 6<br>Other mode of use 7<br>If other, specify………………………………………..	Q199e
How long does the treatment last?	1 to 3 days 1<br>4 to 7 days 2<br>1 week to 1 month 3<br>1 to 3 months 4<br>Several years 5	Q199f
What is the dosage of the treatment?<br>What is the dosage of the treatment?<br>How did you use it?	………………………………………………………………….<br>…………………………………………………………………<br>…………………………………………………………………<br>…………………………………………………………………<br>…………………………………………………………………<br>………………………………………………………………….<br>………………………………………………………………….<br>………………………………………………………………….<br>………………………………………………………………….<br>………………………………………………………………….<br>………………………………………………………………….	Q199g

ID Participant: City: |____| Household: |____|____|____| Participant: |____|____|____|

Are there any prohibitions during the treatment?	Yes 1<br>No 2<br>Don't know/ no answer 77	Q199h
What are the prohibitions during the treatment?	…………………………………………………………….<br>……………………………………………………………<br>……………………………………………………………<br>……………………………………………………………	Q199i

**Did you in the last 21 days:**		
**Question**	**Response**	**Code**
Visit COVID-19 patients?	Yes 1<br>No 2<br>Don't know/ no answer 77	Q200
Work with people with COVID-19?	Yes 1<br>No 2<br>Don't know/ no answer 77	Q201
Have a face-to-face contact with a COVID-19 patient at a distance of less than 1m?	Yes 1<br>No 2<br>Don't know/ no answer 77	Q202
Shared the same enclosed environment as a COVID-19 patient (including sharing a classroom or housekeeping or being at the same gathering)?	Yes 1<br>No 2<br>Don't know/ no answer 77	Q203
Traveled (coach / bus / taxi / personal car / plane) with a COVID-19 patient?	Yes 1<br>No 2<br>Don't know/ no answer 77	Q204
Provided direct care to COVID-19 patients?	Yes 1<br>No 2<br>Don't know/ no answer 77	Q205

ID Participant: City: |____| Household: |____|____|____| Participant: |____|____|____|

2. Clinical Characteristics

1st confirmed case of COVID-19 in Burkina Faso: 03/09/2020
During the past 21 days have you experienced / exhibited any of the following clinical signs:

Question	Response	Code
Fever (≥38 ° C) or history of fever?	Yes 1<br>No 2<br>Don't know/ no answer 77	Q206
Asthenia / fatigue / malaise?	Yes 1<br>No 2<br>Don't know/ no answer 77	Q207
Myalgia / stiffness?	Yes 1<br>No 2<br>Don't know/ no answer 77	Q208
Joint pain?	Yes 1<br>No 2<br>Don't know/ no answer 77	Q209
Headache?	Yes 1<br>No 2<br>Don't know/ no answer 77	Q210
Chills (feeling cold)?	Yes 1<br>No 2<br>Don't know/ no answer 77	Q211
Cough?	Yes 1<br>No 2<br>Don't know/ no answer 77	Q212
Dyspnea (difficulty in breathing)?	Yes 1<br>No 2<br>Don't know/ no answer 77	Q213
Wheezing	Yes 1<br>No 2<br>Don't know/ no answer 77	Q214
Cold?	Yes 1<br>No 2<br>Don't know/ no answer 77	Q215
Epistaxis (nosebleed)?	Yes 1<br>No 2<br>Don't know/ no answer 77	Q216
Sore throat / angina?	Yes 1<br>No 2<br>Don't know/ no answer 77	Q217

ID Participant: City: |____| Household: |____|____|____| Participant: |____|____|____|

Nausea / Vomiting?	Yes 1<br>No 2<br>Don't know/ no answer 77	Q218
Abdominal pain?	Yes 1<br>No 2<br>Don't know/ no answer 77	Q219
Diarrhea?	Yes 1<br>No 2<br>Don't know/ no answer 77	Q220
Conjunctivitis?	Yes 1<br>No 2<br>Don't know/ no answer 77	Q221
Ageusia (loss of taste)?	Yes 1<br>No 2<br>Don't know/ no answer 77	Q222
Anosmia (loss of smell)?	Yes 1<br>No 2<br>Don't know/ no answer 77	Q223
Anorexia (loss of appetite)?	Yes 1<br>No 2<br>Don't know/ no answer 77	Q224
Rash (rashes)?	Yes 1<br>No 2<br>Don't know/ no answer 77	Q225
Disorders of consciousness?	Yes 1<br>No 2<br>Don't know/ no answer 77	Q226
Convulsions?	Yes 1<br>No 2<br>Don't know/ no answer 77	Q227
Other symptoms (to be specified)?<br>……………………………………………	Yes 1<br>No 2<br>Don't know/ no answer 77	Q228
Did you consult because of these symptoms / signs?	Yes 1<br>No 2<br>Don't know/ no answer 77	Q229
Have these symptoms / signs caused you to miss school or work?	Yes 1<br>No 2<br>Don't know/ no answer 77	Q230
Did these symptoms / signs require hospitalization?	Yes 1<br>No 2<br>Don't know/ no answer 77	Q231

ID Participant: City: |____| Household: |____|____|____| Participant: |____|____|____|

3. Venous blood collection		
**Question**	**Response**	**Code**
Blood sample taken?	Yes 1<br>No 2<br>If no, Why? ……………………………………..<br>………………………………………………………………	Q232
Time of sampling?	└─┴─┘ hours └─┴─┘ minutes	Q232a
DBS realized?	Yes 1<br>No 2	Q233
Incident occurred during the sampling?	Yes 1<br>No 2<br>If yes, give details: ……………………………………..<br>………………………………………………………………	Q234

Name and surname of the field investigator Date and signature

ID Participant: City: |____| Household: |____|____|____| Participant: |____|____|____|

4. D63 laboratory results		
**Methods and results of serological tests (a new table for each sample collected)**		
**Question**	**Response**	**Code**
Sample number?	└─┴─┴─┴─┴─┘	Q235
Sampling date?	└─┴─┘ └─┴─┘ └─┴─┴─┴─┘<br>Day/Month/Year	Q236
Time of sampling?	└─┴─┘ hours └─┴─┘ minutes	Q237
Date received at the laboratory?	└─┴─┘ └─┴─┘ └─┴─┴─┴─┘<br>Day/Month/Year	Q238
Reception time at the laboratory?	└─┴─┘ hours └─┴─┘ minutes	Q239
Type of sample?	Serum 1<br>Other type (specify) 2	Q240
Result of the serological test?	IgG ☐ Positive ☐ Negative ☐ Undetermined<br>IgM ☐ Positive ☐ Negative ☐ Undetermined	Q241
Result of the viral load?		Q242
Sample transferred to another laboratory for confirmation?	Yes 1<br>No 2	Q243
if Yes,		
Name and address of the laboratory for confirmation		Q244
When was the sample sent?	└─┴─┘ └─┴─┘ └─┴─┴─┴─┘<br>Day/Month/Year	Q245
When was the result confirmed?	└─┴─┘ └─┴─┘ └─┴─┴─┴─┘<br>Day/Month/Year	Q246
What is the serological test confirmation result?	IgG ☐ Positive ☐ Negative ☐ Undetermined<br>IgM ☐ Positive ☐ Negative ☐ Undetermined	Q247

Name and surname of the biologist

Date and signature
