# Supplementary material for: Incidence rate and predictors of COVID-19 in the two largest cities of Burkina Faso - prospective cohort study in 2021 (ANRS-COV13)
Source: BMC Infect Dis. 2023 Jun 12;23:394. doi: 10.1186/s12879-023-08361-2 (PMC10258776; doi:10.1186/s12879-023-08361-2)
Supplement: Supplementary file 2 — Supplementary Material 2 [file 12879_2023_8361_MOESM2_ESM.docx]

**Incidence rate and predictors of COVID-19 in the two largest cities of Burkina Faso - prospective cohort study in 2021 (ANRS-COV13)**

**Authors:**

Nongodo Firmin KABORE^1^**,** Samiratou Ouédraogo^2,3,4^, Ariane Kamga Mamguem^5^, Isidore Tiandiogo Traoré^1,6^, Dramane Kania^1^, Hermann Badolo^2^, Guillaume Sanou^7^, Amariane Koné^1^, Mimbouré Yara^2^, Thérèse Kagoné^1^, Esperance Ouédraogo^8^, Blahima Konaté^1,9^, Rachel Médah^1^, Nathalie de Rekeneire^10^, Armel Poda^6,11^, Arnaud Eric Diendéré^12^, Boukary Ouédraogo^13^, Oumar Billa^5^, Gilles Paradis^3,4^, Tienhan Sandrine Dabakuyo-Yonli^5^, Halidou Tinto^1,14^

**Affiliations:**

^1^ Centre MURAZ, Institut National de Santé Publique, Bobo-Dioulasso, Burkina Faso.

^2^ Observatoire national de la santé de la population (ONSP), Institut National de Santé Publique, Ouagadougou, Burkina Faso

^3^ Institut National de Santé Publique du Québec (INSPQ), Montréal, Québec, Canada.

^4^ The Department of Epidemiology, Biostatistics and Occupational Health, Faculty of Medicine, McGill University, Montreal, Quebec, Canada.

^5^ Epidemiology and Quality of Life Research Unit, INSERM U1231, Georges Francois Leclerc Centre – UNICANCER, Dijon, France.

^6^ Institut Supérieur des Sciences de la Santé (INSSA), Université Nazi Boni (UNB), Bobo Dioulasso, Burkina Faso.

^7^ Centre National de Recherche et de Formation sur le Paludisme, Institut National de Santé Publique, Ouagadougou, Burkina Faso.

^8^ Département de médicine, pharmacopée traditionnelle et pharmacie, Institut de Recherche en Sciences de la Santé (IRSS) - Centre National de la Recherche Scientifique et Technologique (CNRST), Ouagadougou, Burkina Faso.

^9^ Département de Socio-Économie et d’Anthropologie du Développement (DSEAD), Institut des Sciences des Sociétés (INSS) - Centre National de la Recherche Scientifique et Technologique (CNRST), Ouagadougou, Burkina Faso.

^10^ Fondation Mérieux, Lyon France.

^11^ Service des maladies infectieuses, Centre Hospitalier Universitaire Sourô Sanou, Bobo Dioulasso, Burkina Faso.

^12^ Centre Hospitalier Universitaire de Bogodogo, Ouagadougou, Burkina Faso.

^13^ Direction des systèmes d’information en santé (DSIS), ministère de la Santé et de l’Hygiène Publique, Ouagadougou, Burkina Faso.

^14^ Institut de Recherche en Sciences de la Santé (IRSS) - Unité de Recherche Clinique de Nanoro, Centre National de la Recherche Scientifique et Technologique (CNRST), Burkina Faso.

**Corresponding author**: Nongodo Firmin KABORE

E-mail: [nongodo@yahoo.fr](mailto:nongodo@yahoo.fr)

Address: Centre MURAZ, Institut National de Santé Publique (INSP)

BP 390 Bobo-Dioulasso, Burkina Faso

Phone details: +226 76 66 41 62
