# Supplementary material for: Incidence rate and predictors of COVID-19 in the two largest cities of Burkina Faso - prospective cohort study in 2021 (ANRS-COV13)
Source: BMC Infect Dis. 2023 Jun 12;23:394. doi: 10.1186/s12879-023-08361-2 (PMC10258776; doi:10.1186/s12879-023-08361-2)
Supplement: Supplementary file 3 — Supplementary Material 3 [file 12879_2023_8361_MOESM3_ESM.docx]

**Incidence rate and predictors of COVID-19 in the two largest cities of Burkina Faso - prospective cohort study in 2021 (ANRS-COV13)**

**Additional file 2**

**Table S1.** Distribution of SARS-CoV-2 seronegative participants at baseline, by inclusion in analyses

|  | **Total** | **Included n(%)** | **Not included n(%)** | **p-value** |
| --- | --- | --- | --- | --- |
| **City of residence** | | | | |
| Ouagadougou | 1121 | 921(65.8) | 200(69.4) | 0.237 |
| Bobo-Dioulasso | 566 | 478(34.2) | 88(30.6) |  |
| **Population group** | | | | |
| 10-14 years-old | 236 | 209(14.9) | 27(9.4) | **0.011** |
| 15-18 years-old | 162 | 139(9.9) | 23(8.0) |  |
| Male 19-59 years-old | 409 | 329(23.5) | 80(27.8) |  |
| Female 19-59 years-old | 422 | 358(25.6) | 64(22.2) |  |
| ≥ 60 years-old | 458 | 364(26.0) | 94(32.6) |  |
| **Sex** | | | | |
| Male | 795 | 643(46.0) | 152(52.8) | **0.035** |
| Female | 892 | 756(54.0) | 136(47.2) |  |
| **Age (years)** | | | | |
| 10-18 | 398 | 348(24.9) | 50(17.4) | **0.016** |
| 19-29 | 337 | 274(19.6) | 63(21.9) |  |
| 30-59 | 494 | 413(29.5) | 81(28.1) |  |
| ≥ 60 | 458 | 364(26.0) | 94(32.6) |  |
| **Main occupation during the past 12 months** | | | | |
| Trader/artisan | 470 | 387(31.2) | 83(31.3) | 0.216 |
| Housewife/unemployed | 369 | 294(23.7) | 75(28.3) |  |
| Student | 361 | 309(24.9) | 52(19.2) |  |
| Other | 306 | 251(20.2) | 55(20.8) |  |
| **Educational level** | | | | |
| Not enrolled in school | 450 | 362(26.3) | 88(30.8) | 0.094 |
| Literate/primary | 522 | 437(31.7) | 85(29.7) |  |
| Secondary | 557 | 474(34.4) | 83(29.0) |  |
| University | 136 | 106(7.7) | 30(10.5) |  |
| **≥ 1 exposure to COVID-19 at baseline** | | | | |
| Yes | 41 | 39(3.7) | 2(0.8) | **0.023** |
| No | 1248 | 1012(96.3) | 236(99.2) |  |
| **≥ 1 COVID-19-related symptom at baseline** | | | | |
| Yes | 760 | 630(59.9) | 130(54.6) | 0.132 |
| No | 529 | 421(40.1) | 108(45.4) |  |

**Table S2.** Characteristics of participants by survey

| **Surveys** | **D0** | **D21** | **D42** | **D63** | |
| --- | --- | --- | --- | --- | --- |
| **≥19 years-old** | | | | | |
| **Number of participants** | 1051 | 991 | 773 | 717 | |
| **City of residence, n(%)** | | | | | |
| Ouagadougou | 690(65.7) | 653(65.9) | 512(66.2) | 465(64.9) | |
| Bobo-Dioulasso | 361(34.3) | 338(34.1) | 261(33.8) | 252(35.1) | |
| **Population group, n(%)** | | | | | |
| Male 19-59 yo | 329(31.3) | 307(31.0) | 234(30.3) | 213(29.7) | |
| Female 19-59 yo | 358(34.1) | 349(35.2) | 274(35.4) | 256(35.7) | |
| ≥ 60 yo | 364(34.6) | 335(33.8) | 265(34.3) | 248(34.6) | |
| **Sex, n(%)** | | | | | |
| Male | 511(48.6) | 477(48.1) | 360(46.6) | 332(46.3) | |
| Female | 540(51.4) | 514(51.9) | 413(53.4) | 385(53.7) | |
| **Age (years), n(%)** | | | | | |
| 19-29 | 274(26.1) | 262(26.4) | 199(25.7) | 183(25.5) | |
| 30-59 | 413(39.3) | 394(39.8) | 309(40.0) | 286(39.9) | |
| ≥ 60 | 364(34.6) | 335(33.8) | 265(34.3) | 248(34.6) | |
| **Educational level, n(%)** | | | | | |
| Not enrolled in school | 332(31.6) | 310(31.3) | 230(29.7) | 224(31.2) | |
| Literate/primary | 287(27.3) | 275(27.7) | 210(27.2) | 188(26.2) | |
| Secondary | 326(31.0) | 310(31.3) | 255(33.0) | 230(32.1) | |
| University | 106(10.1) | 96(9.7) | 78(10.1) | 75(10.5) | |
| **Marital status, n(%)** | | | | | |
| Single | 383(36.4) | 357(36.0) | 280(36.2) | 265(37.0) | |
| Coupled | 668(63.6) | 634(64.0) | 493(63.8) | 452(63.0) | |
| **Main occupation during the past 12 months, n(%)** | | | | | |
| Trader/artisan | 380(36.2) | 357(36.0) | 269(34.8) | 247(34.5) | |
| Housewife/unemployed | 290(27.6) | 275(27.7) | 210(27.2) | 201(28.0) | |
| Student | 129(12.3) | 120(12.1) | 101(13.1) | 96(13.4) | |
| Other | 250(23.8) | 237(23.9) | 191(24.7) | 172(24.0) | |
| **Exposure to COVID-19 within the last 14 days, n(%)** | | | | | |
| Yes | 39(3.7) | 12(1.2) | 6(0.8) | 7(1.0) | |
| No | 1012(96.3) | 979(98.8) | 767(99.2) | 710(99.0) | |
| **Symptoms related to COVID-19 within the last 14 days, n(%)** | | | | | |
| Yes | 630(59.9) | 375(37.8) | 234(30.3) | 178(24.8) | |
| No | 421(40.1) | 616(62.2) | 539(69.7) | 539(75.2) | |
| **SARS-CoV-2 serological test results, n(%)** | | | | | |
| Positive | 0(0) | 460(46.4) | 416(53.8) | 413(57.6) | |
| Negative | 1051(100) | 531(53.6) | 357(46.2) | 304(42.4) | |
| **10-18 years-old** | | | | | |
| **Number of participants** |  |  | 348 | | 348 |
| **City of residence, n(%)** | | | | | |
| Ouagadougou |  |  | 231(66.4) | | 231(66.4) |
| Bobo-Dioulasso |  |  | 117(33.7) | | 117(33.7) |
| **Population group, n(%)** | | | | | |
| 10-14 yo |  |  | 209(60.1) | | 209(60.1) |
| 15-18 yo |  |  | 139(39.9) | | 139(39.9) |
| **Sex, n(%)** | | | | | |
| Male |  |  | 132(37.9) | | 132(37.9) |
| Female |  |  | 216(62.1) | | 216(62.1) |
| **Educational level, n(%)** |  |  |  | |  |
| Not enrolled in school |  |  | 30(8.6) | | 30(8.6) |
| Literate/primary |  |  | 150(43.1) | | 150(43.1) |
| Secondary |  |  | 148(42.5) | | 148(42.5) |
| **Main occupation during the past 12 months, n(%)** | | | | | |
| Trader/artisan |  |  | 7(2.0) | | 7(2.0) |
| Housewife/unemployed |  |  | 4(1.1) | | 4(1.1) |
| Student |  |  | 180(51.7) | | 180(51.7) |
| Other |  |  | 1(0.3) | | 1(0.3) |
| **Exposure to COVID-19 within the last 14 days, n(%)** | | | | | |
| Yes |  |  | 4(1.1) | | 4(1.1) |
| No |  |  | 344(98.9) | | 344(98.9) |
| **Symptoms related to COVID-19 within the last 14 days, n(%)** | | | | | |
| Yes |  |  | 95(27.3) | | 95(27.3) |
| No |  |  | 253(72.7) | | 253(72.7) |
| **SARS-CoV-2 serological test results, n(%)** | | | | | |
| Positive |  |  | 0(0) | | 0(0) |
| Negative |  |  | 348(100) | | 348(100) |

Yo: years-old

**Table S3.** Distribution of adult participants (≥19 years-old) by follow-up completeness

|  | **Total** | **Followed to term n(%)** | **Lost to follow up n(%)** | **p-value** |
| --- | --- | --- | --- | --- |
| **Population group** | | | | |
| Male 19-59 years-old | 329 | 213(29.7) | 116(34.7) | 0.163 |
| Female 19-59 years-old | 358 | 256(35.7) | 102(30.5) |  |
| ≥ 60 years-old | 364 | 248(34.6) | 116(34.7) |  |
| **Sex** | | | | |
| Male | 511 | 333 (46.4) | 178 (53.3) | 0.039 |
| Female | 540 | 384(53.6) | 156(46.7) |  |
| **Age (years)** | | | | |
| 19-29 | 274 | 185(25.8) | 89(26.7) | 0.942 |
| 30-59 | 413 | 284(39.6) | 129(38.6) |  |
| ≥ 60 | 364 | 248(34.6) | 116(34.7) |  |
| **Educational level** | | | | |
| Not enrolled in school | 332 | 224(31.2) | 108(32.3) | 0.533 |
| Literate/primary | 287 | 188(26.2) | 99(29.6) |  |
| Secondary | 326 | 230(32.1) | 96(28.7) |  |
| University | 106 | 75(10.5) | 31(9.3) |  |
| **≥ 1 exposure to COVID-19 during follow-up** | | | | |
| Yes | 39 | 27(3.8) | 12(3.6) | 0.890 |
| No | 1012 | 690(96.2) | 322(96.4) |  |
| **≥ 1 COVID-19-related symptom during follow-up** | | | | |
| Yes | 630 | 426(59.4) | 204(61.1) | 0.608 |
| No | 421 | 291(40.6) | 130(38.9) |  |
| **Seroconversion** | | | | |
| Yes | 626 | 459(64.0) | 167(50.0) | <0.001 |
| No | 425 | 258(36.0) | 167(50.0) |  |
