# Supplementary material for: Incidence rate and predictors of COVID-19 in the two largest cities of Burkina Faso - prospective cohort study in 2021 (ANRS-COV13)
Source: BMC Infect Dis. 2023 Jun 12;23:394. doi: 10.1186/s12879-023-08361-2 (PMC10258776; doi:10.1186/s12879-023-08361-2)
Supplement: Supplementary file 4 — Supplementary Material 4 [file 12879_2023_8361_MOESM4_ESM.docx]

**Incidence rate and predictors of COVID-19 in the two largest cities of Burkina Faso - prospective cohort study in 2021 (ANRS-COV13)**

**Additional file 3: Imputation and sensitivity analyses**

*Step 1: assessment of missing data and choice of imputation method*

As shown in **Table 1** of the manuscript, missing data were observed in two variables: “***Main occupation during the past 12 months***” and “***Educational level***”. Almost all the missing data were observed in the 10-18 year age group. All data regarding occupation were missing within the 15-18 years age group (due to an error in the settings of the data collection application) (**Table S4**).

**Table S4**. Distribution of variables with missing data by population group (strata)

|  | **Population group** | | | | |
| --- | --- | --- | --- | --- | --- |
|  | ≥ 60 yo | Males  19-59 yo | Females 19-29 yo | 15-18 yo | 10-14 yo |
| **Main occupation during the past 12 months** | | | | | |
| Student | 0 | 82 | 47 | 0 | 180 |
| Housewife/unemployed | 126 | 11 | 153 | 0 | 4 |
| Trader/artisan | 83 | 169 | 128 | 0 | 7 |
| Other | 153 | 67 | 30 | 0 | 1 |
| Missing data | **2** | **0** | **0** | **139** | **17** |
| **Educational level** | | | | | |
| Not enrolled in school | 195 | 32 | 105 | 19 | 11 |
| Literate/primary | 107 | 81 | 99 | 22 | 128 |
| Secondary | 48 | 152 | 126 | 90 | 58 |
| University | 14 | 64 | 28 | 0 | 0 |
| Missing data | **0** | **0** | **0** | **8** | **12** |

Yo: Years old

**Table S5** presents summary statistics of baseline variables for the complete and the incomplete cases. Data for the occupation variable were missing for all the incomplete cases. We assumed missing data in this study to be missing at random (MAR) i.e. the probability that data are missing does not depend on unobserved data but may depend on observed data. Under MAR, the missing data values do not contain any additional information given observed data about the missing data mechanism (1).

We used multiple imputation (MI), one of the commonly used methods for handling missing data. The way the MI works is that each missing value in the dataset is replaced with an imputed value; this process is repeated with an element of randomness resulting in multiple “completed” datasets, each consisting of observed and imputed values (2).

**Table S5.** Baseline sociodemographic characteristics of participants with complete and incomplete data for the variables in the analysis

| **Variable** | **Complete cases (n=1241)** | **Incomplete cases (n=158)** | **p-value** |
| --- | --- | --- | --- |
| **City of residence** | | | |
| Ouagadougou | 820(66.08) | 101(63.92) | 0.5912 |
| Bobo-Dioulasso | 421(33.92) | 57(36.08) |  |
| **Population group** | | | |
| 10-14 yo | 192(15.47) | 17(10.76) | **<0.001** |
| 15-18 yo | 0 | 139(87.97) |  |
| Male 19-59 yo | 329(26.51) | 0 |  |
| Female 19-59 yo | 358 (28.85) | 0 |  |
| ≥ 60 yo | 362(29.17) | 2(1.27) |  |
| **Sex** | | | |
| Male | 592(47.70) | 51(32.28) | **<0.001** |
| Female | 649(52.30) | 107(67.72) |  |
| **Age (years), median (IQR)** | 38(23-60) | 16(15-18) | **<0.001** |
| **Age group (years)** | | | |
| 10-18 | 192(15.47) | 156(98.73) | **<0.001** |
| 19-29 | 274(22.08) | 0 |  |
| 30-59 | 413(33.28) | 0 |  |
| ≥ 60 | 362(29.17) | 2(1.27) |  |
| **Educational level** | | | |
| Not enrolled in school | 341(27.48) | 21(15.22) | **<0.001** |
| Literate/primary | 412(33.20) | 25(18.12) |  |
| Secondary | 382(30.78) | 92(66.67) |  |
| University | 106(8.54) | 0 |  |
| **Marital status** | | | |
| Single | 381(30.70) | 2(1.27) | **<0.001** |
| Couple | 668(53.83) | 0(0.00) |  |
| Not applicable | 192(15.47) | 156(98.73) |  |
| **Main occupation during the past 12 months** | | | |
| Trader/artisan | 387(31.18) | --- | --- |
| Housewife/unemployed | 294(23.69) | --- |  |
| Student | 309(24.90) | --- |  |
| Other | 251(20.23) | --- |  |

IQR: Interquartile range

*Step 2: Imputation model and choice of variables*

We used multivariate imputation using chained equations (MICE) (1,3). We included all variables from the analysis model in the imputation model to ensure that the imputation model preserved the relationships between the variables of interest (3,4). We also included variables associated with missingness as predictors of missingness in the imputation model (5). As this is a longitudinal database for Cox proportional hazards regression, we include the Nelson-Aalen estimate of the cumulative hazard (HT computed using: sts gen HT = na), as well as the event indicator (_d), which is created with stset command (Stata) in the imputation model (6).

In the absence of a definite rule on the calculation of the number of imputations to be performed, we chose 20, as recommended in the Stata multiple imputation reference manual (1).

**Stata codes we used for the imputation model**

. sts gen HT = na

. mi set flong

. mi register imputed occupation education_level

. mi register regular sex age marital_status city

. mi impute chained (mlogit) occupation (ologit) education_level = HT _d sex age i.marital_status, force augment add(20) by(city) rseed(20231)

*Step 3: Checking the imputation model*

We graphically compared the distribution of occupation (the variable with the most relevant missing values) between the original dataset (observed) and the imputed datasets (imputed). The graphs of the first three imputed datasets are shown.


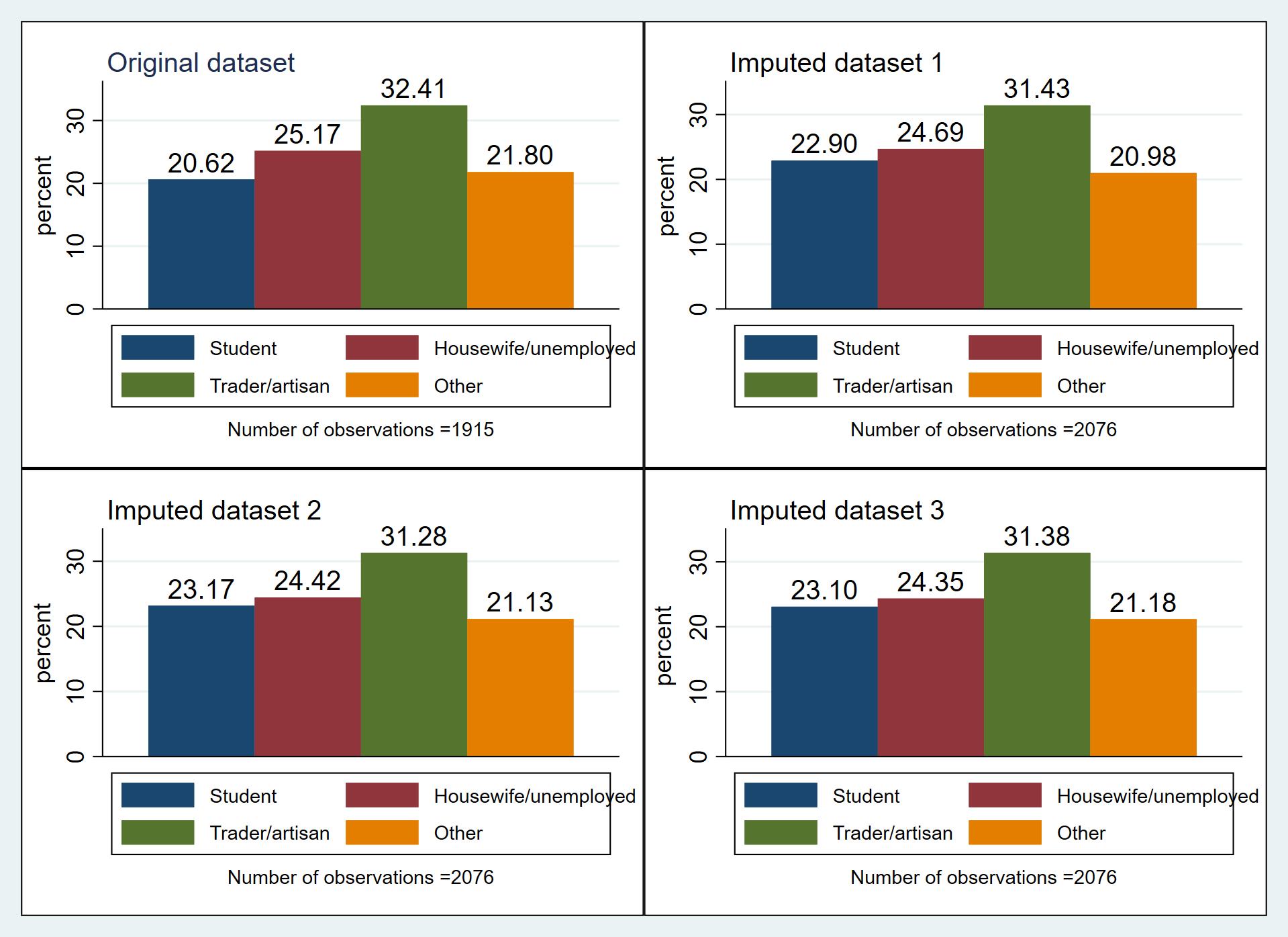


**Figure S1.** Distribution of the “main occupation during the past 12 months” of participants on the original dataset and the first three imputed datasets

*Step 4: analysis models and sensitivity analyses*

In addition to the imputed model, we also constructed two other models for sensitivity analyses. The first was run on the original dataset and the second on the original dataset with missing data replaced by a modality “missing-values”.

**Table S6.** Cox proportional hazards regression for sensitivity analyses

|  | **Model 1*** | | | | **Model 2*** | | | | **Model 3*** | | | |  |
| --- | --- | --- | --- | --- | --- | --- | --- | --- | --- | --- | --- | --- | --- |
|  | **aHR** | | **95%CI** | **p-value** | **aHR** | | **95%CI** | **p-value** | **aHR** | | **95%CI** | **p-value** |  |
| **Age group (years)** | | | | | | | | | | | | |  |
| 10-18 (ref.) | 1 | | --- | --- | 1 | | --- | --- | 1 | | --- | --- |  |
| 19-29 | **2.06** | | [1.42 – 2.99] | **<0.001** | **1.96** | | [1.36 - 2.83] | **<0.001** | **1.71** | | [1.27 - 2.30] | **<0.001** |  |
| 30-59 | **2.21** | | [1.46 - 3.36] | **<0.001** | **2.09** | | [1.39 - 3.14] | **<0.001** | **1.85** | | [1.31 - 2.62] | **0.001** |  |
| ≥ 60 | **1.88** | | [1.22 - 2.89] | **0.004** | **1.76** | | [1.16 - 2.67] | **0.008** | **1.57** | | [1.09 - 2.25] | **0.015** |  |
| **Main occupation during the past 12 months** | | | | | | | | | | | | |  |
| Student (ref.) | 1 | | --- | --- | 1 | | --- | --- | 1 | | --- | --- |  |
| Housewife/unemployed | 1.09 | | [0.81 - 1.48] | 0.573 | 1.11 | | [0.82 - 1.51] | 0.486 | 1.07 | | [0.79 - 1.44] | 0.680 |  |
| Trader/artisan | 1.15 | | [0.86 - 1.55] | 0.342 | 1.17 | | [0.88 - 1.57] | 0.284 | 1.13 | | [0.84 - 1.51] | 0.413 |  |
| Other | 1.17 | | [0.84 - 1.62] | 0.349 | 1.20 | | [0.87 - 1.65] | 0.281 | 1.14 | | [0.83 - 1.58] | 0.414 |  |
| Missing values modality | --- | | --- | --- | 1.43 | | [0.95 - 2.17] | 0.091 | --- | | --- | --- |  |
|  | | | | | | | | | | | | |  |
| ***Number of observations*** | 1915 | | | | 2076 | | | | 2076 | | | |  |
| ***Akaike information criterion (AIC)*** | | 8090.666 | | | | 8787.616 | | | | --- | | | |
| ***Bayesian information criterion (BIC)*** | | 8124.011 | | | | 8827.083 | | | | --- | | | |

aHR : adjusted hazard ratio

*Model 1: Analysis on the original dataset (with missing values)

*Model 2: Analysis on the original dataset with missing data replaced by a modality “missing-values”

*Model 3: Analysis on multiple imputed datasets

**References**

1. Stata Bookstore | Multiple-Imputation Reference Manual, Release 18 [Internet]. [cited 2023 May 21]. Available from: https://www.stata.com/bookstore/multiple-imputation-reference-manual/

2. Rubin DB. Frontmatter. In: Multiple Imputation for Nonresponse in Surveys [Internet]. John Wiley & Sons, Ltd; 1987 [cited 2023 May 21]. p. i–xxix. Available from: https://onlinelibrary.wiley.com/doi/abs/10.1002/9780470316696.fmatter

3. White I, Royston P, Wood A. White IR, Royston P, Wood AMMultiple imputation using chained equations: Issues and guidance for practice. Stat Med 30(4): 377-399. Stat Med. 2011 Feb 20;30:377–99.

4. Knorr-Held L. Analysis of Incomplete Multivariate Data. J. L. Schafer, Chapman & Hall, London, 1997. No. of pages: xiv+430. Price: £39.95. ISBN 0-412-04061-1. Stat Med. 2000;19(7):1006–8.

5. Sterne JAC, White IR, Carlin JB, Spratt M, Royston P, Kenward MG, et al. Multiple imputation for missing data in epidemiological and clinical research: potential and pitfalls. BMJ. 2009 Jun 29;338:b2393.

6. White IR, Royston P. Imputing missing covariate values for the Cox model. Stat Med. 2009;28(15):1982–98.
